# Adipocyte inflammation is the primary driver of hepatic insulin resistance in a human iPSC-based microphysiological system

**DOI:** 10.1038/s41467-024-52258-w

**Published:** 2024-09-12

**Authors:** Lin Qi, Marko Groeger, Aditi Sharma, Ishan Goswami, Erzhen Chen, Fenmiao Zhong, Apsara Ram, Kevin Healy, Edward C. Hsiao, Holger Willenbring, Andreas Stahl

**Affiliations:** 1 Department of Nutritional Science and Toxicology, College of Natural Resources, University of California Berkeley, Berkeley, CA 94720 USA; 2 Division of Transplant Surgery, Department of Surgery, University of California San Francisco, San Francisco, CA 94143 USA; 3 Eli and Edythe Broad Center for Regeneration Medicine, University of California San Francisco, San Francisco, CA 94143 USA; 4 Division of Endocrinology and Metabolism, Department of Medicine, University of California San Francisco, San Francisco, CA 94143 USA; 5 Institute for Human Genetics, University of California San Francisco, San Francisco, CA 94143 USA; 6 Department of Bioengineering, College of Engineering, University of California Berkeley, Berkeley, CA 94720 USA; 7 Department of Materials Science and Engineering, College of Engineering, University of California Berkeley, Berkeley, CA 94720 USA; 8 Liver Center, University of California San Francisco, San Francisco, CA 94143 USA

**Keywords:** Stem-cell biotechnology, Type 2 diabetes, Obesity, Lab-on-a-chip, Non-alcoholic fatty liver disease

## Abstract

Interactions between adipose tissue, liver and immune system are at the center of metabolic dysfunction-associated steatotic liver disease and type 2 diabetes. To address the need for an accurate in vitro model, we establish an interconnected microphysiological system (MPS) containing white adipocytes, hepatocytes and proinflammatory macrophages derived from isogenic human induced pluripotent stem cells. Using this MPS, we find that increasing the adipocyte-to-hepatocyte ratio moderately affects hepatocyte function, whereas macrophage-induced adipocyte inflammation causes lipid accumulation in hepatocytes and MPS-wide insulin resistance, corresponding to initiation of metabolic dysfunction-associated steatotic liver disease. We also use our MPS to identify and characterize pharmacological intervention strategies for hepatic steatosis and systemic insulin resistance and find that the glucagon-like peptide-1 receptor agonist semaglutide improves hepatocyte function by acting specifically on adipocytes. These results establish our MPS modeling the adipose tissue-liver axis as an alternative to animal models for mechanistic studies or drug discovery in metabolic diseases.

## Abstract (web summary)

In vitro modelling of the adipose tissue-liver axis can advance understanding and therapy of metabolic disease, including by distinguishing effects of obesity and inflammation. Here, authors develop such a system based on isogenic human iPSCs and interconnected microphysiological devices.

## Introduction

The steadily increasing incidence of obesity, affecting ~ 40% of adults in the US^1^, urges for a better mechanistic understanding of human obesity-associated metabolic diseases and the development of effective and safe treatments^2^. Increased visceral adipose tissue mass is linked to multiple disease conditions, including type 2 diabetes mellitus (T2DM) and metabolic dysfunction-associated steatotic liver disease (MASLD)^3–6^. Although obesity is strongly correlated with the risk of MASLD development, not all obese individuals develop metabolic dysfunctions, highlighting the need for comprehensive studies of disease triggers^7^.

Expansion of white adipose tissue (WAT) is frequently associated with inflammation caused by accumulation of tissue-resident activated (M1) macrophages^8^. WAT inflammation leads to dysregulated lipolysis, insulin resistance, increased secretion of proinflammatory cytokines and altered secretion of adipokines, all of which affect other organs, including the liver^2^. Whether WAT expansion or its inflammation is the main trigger in WAT-driven MASLD and hepatic insulin resistance (HIR) has been difficult to address in animal models because lipid accumulation and lipotoxic events often go hand in hand with inflammation of WAT and liver^9–15^. The clinical relevance of data from conventional in vitro models in which cellular monocultures are challenged with fatty acids or proinflammatory cytokines is also limited^9,12,16^. Therefore, there is a need for advanced human-specific in vitro models that address the complexity of obesity-induced HIR and define the role of WAT expansion vs inflammation.

Microphysiological systems (MPS) are superior to conventional in vitro models in mimicking human liver tissue^17–20^ and WAT^21–23^ by allowing for increased cellular complexity, 3D tissue structure and dynamic culture conditions. These characteristics proved to be instrumental for modeling of MASLD using hepatic cell lines^24–26^, primary cells^16,27–29^ and human induced pluripotent stem cell (iPSC)-derived cells^30,31^.

Multi-tissue MPS are particularly clinically relevant because they address the importance of tissue interaction in metabolism. A pioneering study identified adipocyte-derived factors that cause metabolic alterations in hepatocytes in a primary human cell-based WAT-liver MPS, including under culture conditions mimicking T2DM and obesity^32^. Similarly, media transfer experiments between murine adipocytes and hepatocytes showed that secreted factors act as mediators of communication between WAT and liver to regulate lipid metabolism^33^. Although primary cells are considered the gold-standard for disease modeling and drug testing, their limited availability, variable quality and genetic heterogeneity represent substantial hurdles for the development of multi-tissue MPS.

Recent protocols can produce iPSC-derived hepatocytes (iHEPs), adipocytes (iADIPOs) and macrophages (iMACs) that closely resemble primary cells in differentiation and function^8,34–36^. Here, we use iADIPOs and iHEPs to develop an interconnected WAT-liver MPS, including modeling of WAT inflammation by exposure to proinflammatory M1-iMACs. We show that this isogenic iPSC-based MPS allows for dissecting the effects of WAT expansion and inflammation on metabolic hepatocyte function and rational screening for MASLD drugs.

## Results

### Functional characterization and scaling of iADIPO-MPS and iHEP-MPS

**Fig. 1:**
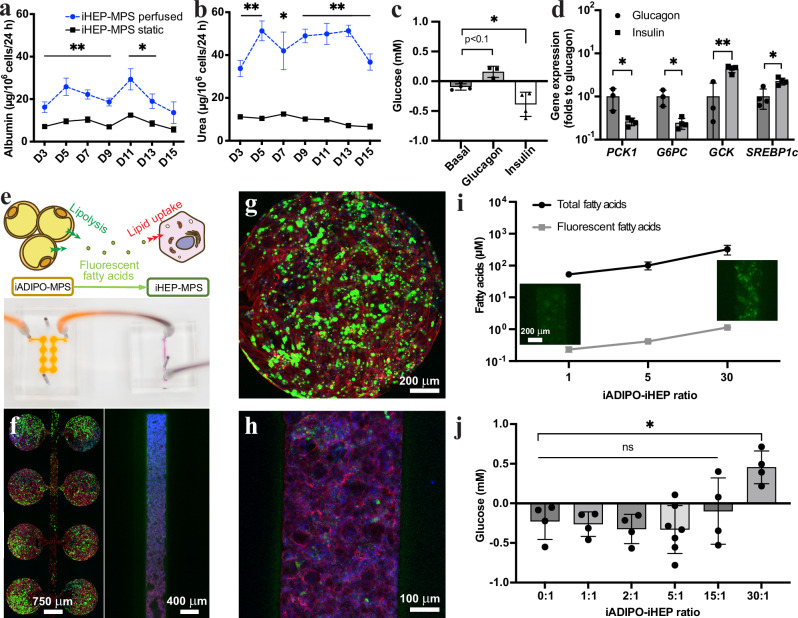
Interconnection and scaling of iHEP-MPS and iADIPO-MPS. **a**, **b** Albumin (**a**) and urea (**b**) in media of iHEP-MPS under perfused (blue dashed line) and static (black solid line) conditions for 15 days. *n* = 14, 12, 12, 14, 5, 10, 5 in the perfused conditions and *n* = 13, 14, 14, 11, 5, 11, 7 in the static conditions for D3-D15 in (**a**). *n* = 3 in all conditions in (**b**). *n* referring to media collected from both conditions. *p*-values were calculated by unpaired two-tailed *t*-tests for the comparison of perfused vs static each day. **c**, **d** Glucose in media (**c**) (*n* = 4 for Basal, Insulin; *n* = 3 for Glucagon) and metabolic gene expression in iHEPs (**d**) (*n* = 4 except *n* = 3 for Glucagon *PCK1, G6PC, GCK*) of iHEP-MPS after 1 h hormonal stimulation. n referring to media in (**c**) and MPS in (**d**). *p*-values were calculated by one-way ANOVA followed by Tukey’s test in (**c**) and by unpaired two-tailed *t*-tests in (**d**). **e** Schematic and photo of interconnected iADIPO-MPS and iHEP-MPS. **f**–**h** Representative fluorescent images of iADIPO-MPS (left image in **f**, **g**) and iHEP-MPS (right image in **f**, **h**). Blue, nuclei; red, F-actin; green, fatty acids. **i** Total non-esterified fatty acid and fluorescent fatty acid concentrations in media at different iADIPO-iHEP ratios. Insets show representative fluorescent images of iHEP-MPS at 1:1 and 30:1 ratios. *n* = 3 except *n* = 4 for Total fatty acids 1,5. *n* referring to media. Two-tailed correlation test shows Pearson *r* = 0.9996 and *p* = 0.0173 between total and fluorescent fatty acids. **j** Glucose levels in media of 1 h insulin-stimulated iHEP-MPS 48 h after interconnection with iADIPO-MPS at different cell ratios. *n* = 4 except *n* = 7 for 5:1. *n* referring to media. *p*-values were calculated by one-way ANOVA then Dunnett’s test. All data are mean ± SD. *p < 0.05; **p < 0.005; ns, *p* > 0.05. Statistical methods and exact *p*-values are shown in Supplementary Data 1. Source data are provided as Source Data file.

We determined the effects of MPS culture on iHEPs generated with a protocol designed to optimize metabolic function^34^. For this, we loaded the cells into an MPS designed as previously reported^37^. We found that iHEPs benefit from MPS culture as evidenced by higher albumin and urea secretion over 15 days compared to conventional static culture (Fig. 1a, b). In addition, the iHEP-MPS showed physiological responses to insulin and glucagon of hepatic glucose production (HGP) and glucose and lipid metabolism-associated gene expression (Fig. 1c, d).

Next, we investigated whether iADIPO-iHEP co-culture using a transwell system allows for analysis of lipid uptake and release dynamics (Supplementary Fig. 1a). For this, we cultured iADIPOs in 24-well plates and preloaded them with fluorescent fatty acids for 24 h, after which we co-cultured them with iHEPs in transwell inserts for 24 h. We found increased fatty acid uptake by iHEPs after induction of lipolysis with isoproterenol in iADIPOs (Supplementary Fig. 1b, c). Moreover, we found impaired insulin-mediated reduction of glucose output by iHEPs under lipolysis-inducing conditions when we increased the iADIPO-iHEP ratio to 5:1; HGP continued to be insulin responsive under basal conditions, i.e., without induction of lipolysis (Supplementary Fig. 1d, e).

Next, we developed an interconnected iADIPO-iHEP MPS allowing for scalable cell ratios based on our previously reported iADIPO-MPS design^8^ (Fig. 1e, f). We investigated iADIPO-iHEP ratios ranging from 1:1 to 30:1 under basal conditions. To reliably quantify lipid accumulation in iHEPs, we first analyzed absorption of fluorescent fatty acids by the PDMS used to build the iHEP-MPS and iADIPO-MPS, which showed no bias for up to 4 days (Supplementary Fig. 2a). We found that preloaded fluorescent fatty acids were spontaneously released from the iADIPO-MPS into the flow-through media and transferred to the iHEP-MPS (Fig. 1g, h). Both total and fluorescent fatty acids in iHEPs increased as a linear function of iADIPO number (Pearson *R* > 0.99 and *p*-value < 0.05), which confirmed the utility of fluorescent fatty acid uptake as a proxy for total fatty acid accumulation (Fig. 1i). Analysis of insulin regulation of HGP after 48 h of iADIPO-iHEP-MPS interconnection showed insulin resistance only at the highest iADIPO-iHEP ratio of 30:1 (Fig. 1j). Because this ratio is unphysiologically high^38^, our findings suggest that accumulation of metabolically healthy WAT mass alone is not sufficient to drive HIR and its hepatic complications.

### Inflamed iADIPO-MPS causes steatosis and insulin resistance in iHEP-MPS

**Fig. 2:**
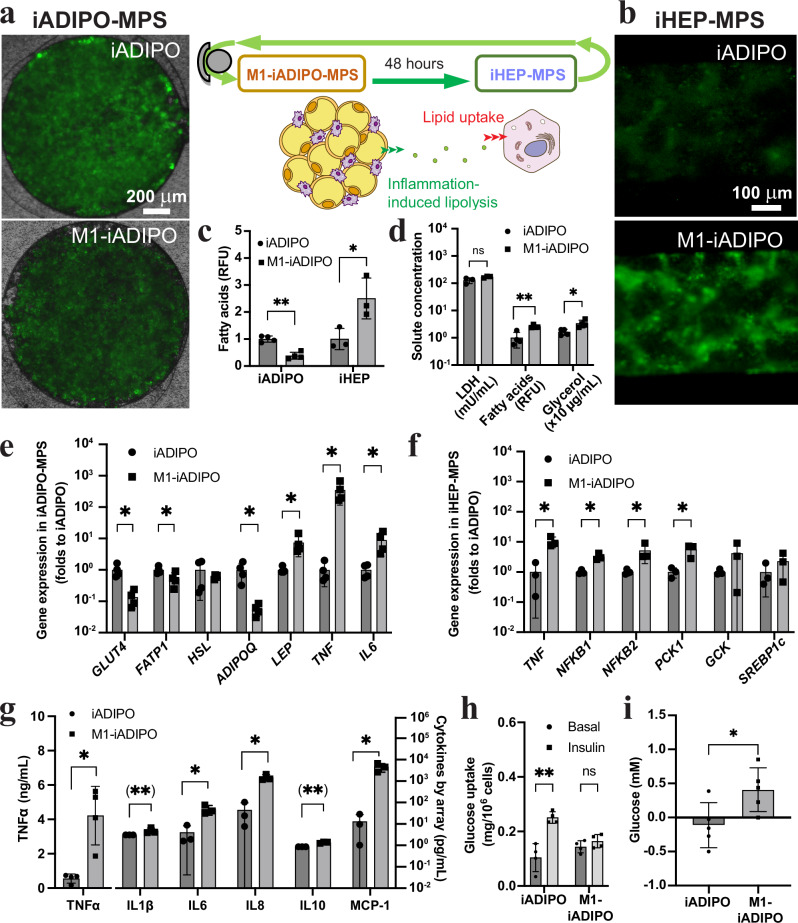
Modeling the link between WAT inflammation and MASLD in iADIPO-iHEP-MPS. **a**–**c** Schematic of iADIPO-MPS and iHEP-MPS interconnection and representative images and quantification of fatty acids in iADIPO-MPS (**a**–**c**) and iHEP-MPS (**b**, **c**). M1-iADIPO, iADIPO-MPS containing M1-iMACs; iADIPO, iADIPO-MPS without iMACs. *n* = 4 in iADIPO and *n* = 3 in iHEP. *n* referring to media. **d** LDH and lipolysis products in circulating media. *n* = 4 except *n* = 3 for LDH. *n* referring to media. **e**, **f** Gene expression in iADIPO-MPS (**e**) (*n* = 4) and iHEP-MPS (**f**) (*n* = 3 except *n* = 4 for iADIPO *NFKB2*). *n* referring to MPS. **g** Cytokine levels in circulating media. *n* = 3 except *n* = 4 for TNFα. n referring to media. (**) indicates values that were set to detection limit of the assay. **h** Glucose uptake in iADIPO-MPS measured by the clearance of glucose in the assay media. *n* = 4 referring to media. **i** Glucose levels in media of 1 h insulin-stimulated iHEP-MPS. *n* = 5 referring to media. All data are mean ± SD. *p*-values were determined by paired two-tailed *t*-test in (**h**) and unpaired two-tailed *t*-test elsewhere. *p < 0.05; **p < 0.005; ns, *p* > 0.05. Statistical methods and exact *p*-values are shown in Supplementary Data 1. Source data are provided as Source Data file.

Recently, we showed that activated proinflammatory M1-iMACs cause insulin resistance independent of steatosis in iHEPs in transwell culture^34^. To determine the effect of WAT inflammation on glucose and lipid metabolism in hepatocytes, we introduced M1-iMACs into iADIPO-MPS for 24 h at an M1-iMAC-iADIPO ratio of 1:10 before interconnection to iHEP-MPS at an iADIPO-iHEP ratio of 5:1. After 48 h of interconnection, the inflamed M1-iADIPO-MPS showed decreased intracellular uptake of fluorescent fatty acids compared to the iADIPO-MPS cultured without iMACs (Fig. 2a–c), reflecting higher lipolytic activity (Fig. 2d). Consequently, the iHEP-MPS showed increased fatty acid uptake and lipid accumulation under inflamed iADIPO-MPS conditions (Fig. 2b, c). As expected, inflammation markers were increased in the M1-iADIPO-MPS, including gene expression of the proinflammatory cytokine *TNF*. Key adipokines were also altered, with increased gene expression of *LEP*, which encodes leptin and inhibits lipogenesis and increases lipolysis, and decreased gene expression of *ADIPOQ*, which encodes insulin-sensitizing adiponectin (Fig. 2e). Similarly, the iHEP-MPS showed increased expression of the inflammatory genes *TNF* and *NFKB1/2* (Fig. 2f). In accordance, we found higher levels of proinflammatory cytokines in the media circulating between the iADIPO-MPS and iHEP-MPS (Fig. 2g). Analysis of metabolic effects revealed decreased expression of the insulin-responsive glucose and fatty-acid transporter genes *GLUT4* and *FATP1* in the iADIPO-MPS under inflammatory conditions (Fig. 2e), whereas *PCK1* gene expression was increased in the iHEP-MPS, suggesting activation of gluconeogenesis and development of insulin resistance (Fig. 2e, f). We confirmed this finding by analysis of insulin-mediated glucose uptake in the iADIPO-MPS (Fig. 2h) and HGP in the iHEP-MPS (Fig. 2i), both of which were pathologically altered by inflammation compared to the control condition. In contrast, the addition of non-activated (M0) iMACs to the iADIPO-MPS neither increased TNFα secretion nor disrupted insulin-mediated regulation of glucose uptake in the iADIPO-MPS or HGP in the iHEP-MPS, similar to control conditions without iMACs (Supplementary Fig. 3a–c).

To determine the contribution of adipocytes to the alterations in glucose and lipid metabolism in hepatocytes caused by WAT inflammation, we interconnected iHEP-MPS with MPS only containing M1-iMACs and compared the results to iHEP-MPS interconnected with M1-iADIPO-MPS (Supplementary Fig. 3d). We found that inclusion of iADIPOs worsened the metabolic alterations in iHEP-MPS, including higher HGP, earlier onset of insulin resistance, higher lipid levels and higher expression of genes reflecting inflammation, gluconeogenesis, lipogenesis and lipid transport (Supplementary Fig. 3e–h). These findings show that alterations of adipocytes caused by proinflammatory macrophages disrupt glucose and lipid metabolism in hepatocytes.

### Rational drug screening in iADIPO-iHEP-MPS reveals distinct effects on inflammation and lipid and glucose metabolism

**Fig. 3:**
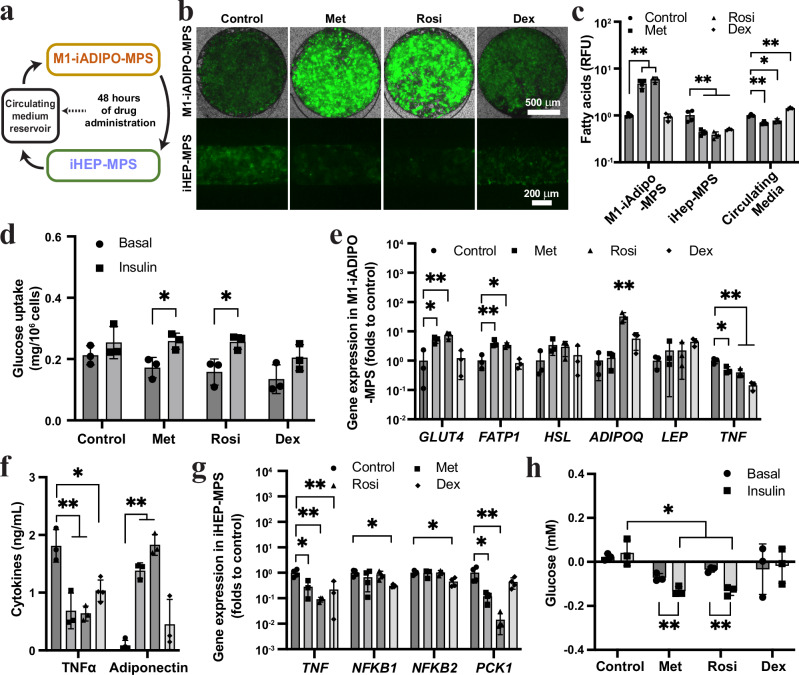
Drugs normalizing inflammation and glucose and lipid metabolism in M1-iADIPO-iHEP-MPS. **a** Schematic of MPS interconnection and drug administration. **b**, **c** Representative images (**b**) and quantification (**c**) of fluorescent fatty acids (green) in M1-iADIPO-MPS and iHEP-MPS. Met, metformin; Rosi, rosiglitazone; Dex, dexamethasone. *n* = 4 for M1-iADIPO-MPS except *n* = 3 for Rosi. *n* = 4 for iHEP-MPS except *n* = 3 for Dex. *n* = 3 for circulating media. *n* referring to MPS. **d** Glucose uptake in M1-iADIPO-MPS measured by the clearance of glucose in the assay media. *n* = 3 referring to media. **e** Gene expression in iMAC-iADIPO-MPS. *n* = 3 referring to MPS. **f** TNFα and adiponectin levels in circulating media. *n* = 3 except *n* = 4 for TNFα-Dex and Adiponectin-Control. n referring to media. **g** Gene expression in iHEP-MPS. *n* = 4 except *n* = 3 for Met, Rosi, Dex *TNF* and Met, Rosi *NFKB2*. n referring to MPS. **h** Glucose levels in media of iHEP-MPS without and with 1 h insulin-stimulation. *n* = 3 referring to media. All data are mean ± SD. *p*-values were determined by paired two-tailed *t*-tests in Basal vs Insulin comparison for each drug in (**d**) and (**h**), by one-way ANOVA followed by Tukey’s test in multiple comparisons for each group in (**c**–**g**) and two-way ANOVA followed by Tukey’s test for cross-group comparison in (**h**). *p < 0.05; **p < 0.005. Statistical methods and exact *p*-values are shown in Supplementary Data 1. Source data are provided as Source Data file.

To demonstrate the utility of our MPS representing the WAT-liver axis for drug discovery, we selected two well-recognized insulin sensitizers (metformin at 10 mM and rosiglitazone at 1 μM) and one anti-inflammatory drug (dexamethasone at 0.5 μM) to assess their efficacy in treating obesity-induced MASLD/T2DM in the M1-iADIPO-iHEP-MPS (Fig. 3a). We analyzed drug effects 48 h after MPS connection and excluded drug absorption by PDMS at this time point (Supplementary Fig. 2b). We found that all of these drugs reduced lipid accumulation in iHEPs (Fig. 3b, c). Metformin and rosiglitazone were equally effective in increasing intracellular fatty acids in iADIPOs, resulting in lower amounts of fatty acids circulating in the media, and normalization of insulin-stimulated glucose uptake in the iADIPO-MPS (Fig. 3c, d). Dexamethasone had no effect on fatty acid or glucose uptake in the iADIPO-MPS (Fig. 3c, d). These findings were consistent with drug-induced changes in *GLUT4* and *FATP1* gene expression observed in the iADIPO-MPS (Fig. 3e). All of the drugs decreased *TNF* gene expression in the iADIPO-MPS; they all also decreased secretion of TNFα and increased secretion of adiponectin into the media (Fig. 3e, f). However, only metformin and rosiglitazone decreased *TNF* and *PCK1* gene expression in the iHEP-MPS and improved insulin response (Fig. 3g, h). These findings show that metformin and rosiglitazone prevent inflammation-induced dysfunction of lipid and glucose metabolism in WAT and hepatocytes.

### Adipose tissue-specific incretin prevents MASLD

Treatment of obese patients with glucagon-like peptide-1 receptor (GLP1R) agonists has shown hepatoprotective effects^39^. However, the specific mechanisms of action in the liver are incompletely understood. Moreover, in contrast to other metabolically active cells such as adipocytes^40,41^, whether GLP1R is expressed at relevant levels and metabolically active in hepatocytes is controversial^42^. Therefore, we determined whether the GLP1R agonist semaglutide (at 1 μM) acts differently on iADIPOs, iHEPs and iMACs in separate MPS or conventional monocultures before testing whether it can improve metabolic function in our M1-iADIPO-iHEP-MPS (Supplementary Fig. 4). We found that only the iADIPO-MPS responded to semaglutide. Gene expression changes in the iADIPO-MPS included increased expression of *GLUT4* and *HSL*, suggesting higher glucose clearance and lipolysis (Supplementary Fig. 4a, b). We also found increased gene expression of the general WAT marker *PPARG* and the subcutaneous WAT marker *PNPLA3* as well as *ADIPOQ*, which translated into increased secretion of adiponectin into the media (Supplementary Fig. 4a–c). In accordance, *GLP1R* gene expression was significantly higher in iADIPOs than iHEPs and iMACs, matching its expression levels in primary human WAT and primary human hepatocytes (Supplementary Fig. 5a and b). None of the iPSC-derived cell types showed a difference in *TNF* gene expression and TNFα secretion was not altered by semaglutide treatment in iADIPO-MPS or M1-iMAC monocultures (Supplementary Fig. 4a, b, d–f).

**Fig. 4:**
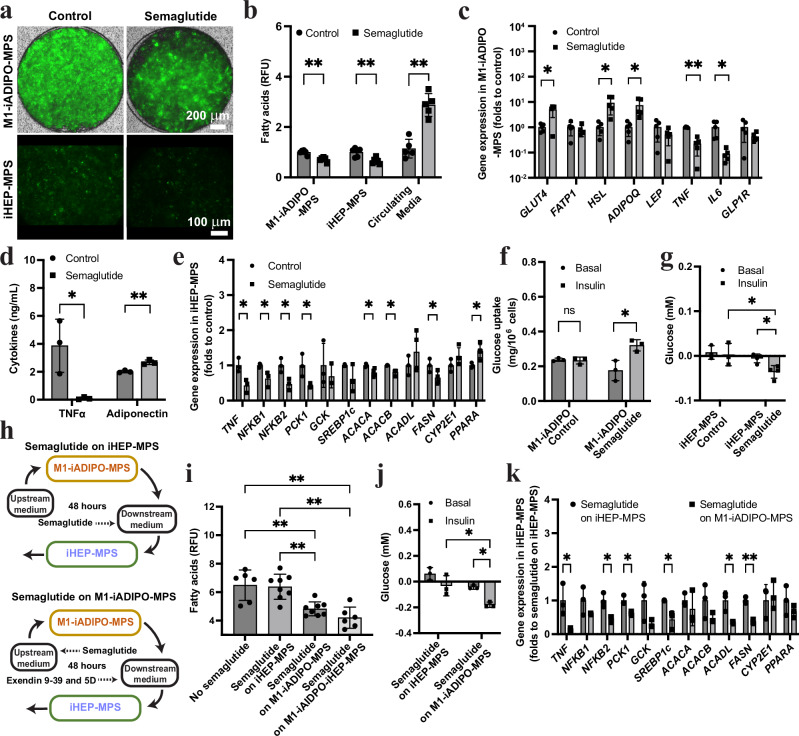
Identification of the effects of semaglutide on WAT inflammation-induced MASLD in M1-iADIPO-iHEP-MPS. **a**, **b** Representative images (**a**) and quantification (**b**) of fluorescent fatty acids in M1-iADIPO-MPS and iHEP-MPS. *n* = 6 except *n* = 5 for circulating media. *n* referring to MPS. **c** Gene expression in iMAC-iADIPO-MPS. *n* = 5 referring to MPS. **d** TNFα and adiponectin levels in circulating media. *n* = 3 referring to media. **e** Gene expression in iHEP-MPS. *n* = 3 except *n* = 4 for *ACACA* to *CYP2E1* and *n* = 5 for Semaglutide *PPARA*. n referring to MPS. **f** Glucose uptake in M1-iADIPO-MPS in response to insulin. *n* = 3 referring to media. **g** Glucose levels in media of iHEP-MPS without and with 1 h insulin-stimulation. *n* = 3 for Control and *n* = 4 for Semaglutide. *n* referring to MPS. **h** Schematic of selective semaglutide treatment of iHEP-MPS (top) or M1-iADIPO-MPS, the latter including semaglutide antagonism with exendin 9–39 and compound 5D in iHEP-MPS (bottom). **i**, **j** Quantification of fatty acids (**i**) (*n* = 8 except *n* = 6 for No semaglutide and Semaglutide on M1-iADIPO-iHEP-MPS. *n* referring to MPS) and glucose without and with 1 h insulin-stimulation in media of iHEP-MPS (**j**) (*n* = 3 referring to media). **k** Gene expression in iHEP-MPS. *n* = 3 referring to MPS. All data are mean ± SD. *p*-values were determined by paired two-tailed *t*-test in Basal vs Insulin comparison in (**f**, **g**–**j**), one-way ANOVA followed by Tukey’s test in (**i**), two-way ANOVA followed by Tukey’s test in cross-group comparison in (**g**, **j**) and by unpaired two-tailed *t*-test elsewhere. *p < 0.05; **p < 0.005; ns, *p* > 0.05. Statistical methods and exact *p*-values are shown in Supplementary Data 1. Source data are provided as Source Data file.

To determine whether the observed WAT-specific effects of semaglutide improve metabolic function of hepatocytes, we treated M1-iADIPO-iHEP-MPS with semaglutide (at 1 μM) in the circulating media for 48 h. We observed increased lipolysis in the iADIPO-MPS, as evidenced by reduced fluorescent intensity and increased fatty acids in the circulating media (Fig. 4a, b), which was accompanied by increased *HSL* gene expression (Fig. 4c). Surprisingly, lipid accumulation in the iHEP-MPS also decreased after semaglutide treatment compared to the untreated control (Fig. 4a, b). These changes were accompanied by significantly higher *GLUT4* and *ADIPOQ* gene expression in the iADIPO-MPS, increased secretion of adiponectin into the media, and overall reduced inflammatory activity (Fig. 4c, d). Foremost, TNFα secretion from the iADIPO-MPS into the circulating media decreased 30-fold (Fig. 4d). In accordance, after semaglutide treatment the iHEP-MPS showed decreased expression of the inflammatory genes *TNF* and *NFKB1/2*, which was accompanied by reduced *PCK1* gene expression (Fig. 4e). Expression of the lipogenesis-associated genes *ACACA*, *ACACB* and *FASN* was also decreased, whereas expression of *PPARA* was increased in the iHEP-MPS, suggesting increased β-oxidation, together explaining our finding of reduced lipid accumulation in the iHEP-MPS after semaglutide treatment (Fig. 4a, b, e). In addition, semaglutide improved insulin sensitivity, resulting in restoration of insulin-mediated glucose uptake in the iADIPO-MPS and insulin-mediated suppression of HGP in the iHEP-MPS (Fig. 4f, g), probably mediated by its anti-inflammatory effect reducing TNFα levels^8,34^.

To confirm the tissue-specific effects of semaglutide in the M1-iADIPO-iHEP-MPS, we employed a non-circulating flow-through interconnection to selectively stimulate the M1-iADIPO-MPS or the iHEP-MPS component (Fig. 4h). For this, we added semaglutide (at 1 μM) for 48 h either after or before the M1-iADIPO-MPS component. We used the latter configuration in combination with the semaglutide antagonists exendin 9-39 (at 1 μM) and compound 5D (at 1 μM) (Fig. 4h). While selective semaglutide treatment of the M1-iADIPO-MPS component was as effective as treatment of the entire M1-iADIPO-iHEP-MPS, selective semaglutide treatment of the iHEP-MPS component was largely ineffective as evidenced by higher lipid accumulation and impaired insulin-mediated suppression of HGP (Fig. 4i, j). Furthermore, gene expression markers of inflammation, gluconeogenesis and lipogenesis were higher in the iHEP-MPS-targeting condition compared to the condition targeting the M1-iADIPO-MPS (Fig. 4k). These findings show that semaglutide prevents lipid accumulation and insulin resistance in hepatocytes by reducing WAT inflammation.

## Discussion

The WAT-liver axis maintains metabolic homeostasis and plays an important role in driving metabolic dysfunction in obese patients^43^. To model the interaction of WAT and hepatocytes in the development of MASLD, we developed an iPSC-based multi-tissue MPS that not only allows for upscaling of the WAT component but also considers inflammation as a disease driver.

Consistent with our previous results^8,34^ and human studies^44^, our model shows that M1-iMAC-mediated inflammation readily induces metabolic dysfunction, including increased WAT lipolysis and induction of systemic insulin resistance. In contrast, we found that the cellular ratio of iADIPOs to iHEPs needs to be escalated to 30:1 to induce HIR in the absence of M1-iMAC-mediated inflammation. Given that ratios of adipocytes to hepatocytes range from 1:10 in lean to 1:1 in obese humans^38,45^, our findings show that healthy WAT causes metabolic dysfunction in hepatocytes only if the mass is expanded to clinically irrelevant levels. Therefore, our findings suggest that WAT inflammation, rather than mere WAT expansion, is required for the development of MASLD in obese individuals^7^.

To further understand the relationship of inflammation and steatosis, we tested how our model responds to drugs clinically used for T2DM and MASLD therapy. We focused on metformin, which is a widely used insulin sensitizer reported to promote AMPK activation and inhibit mitochondrial respiration^46^, and rosiglitazone, a PPARγ agonist that increases adipogenesis^47^. In addition to these insulin-sensitizing drugs, we tested dexamethasone because of its anti-inflammatory properties^48^. Although dexamethasone was effective in reducing inflammatory markers in the M1-iADIPO-iHEP-MPS, it failed to improve insulin sensitivity, probably because of its known insulin-desensitizing effects on adipocytes and hepatocytes^35,49,50^. Metformin and rosiglitazone showed comparable beneficial effects on lipolysis, inflammation and insulin resistance in the iADIPO-MPS, but rosiglitazone was more effective than metformin in reversing inflammation and insulin resistance in the iHEP-MPS. These findings suggest superior efficacy of rosiglitazone against MASLD, particularly when considering the high metformin concentration used here as compared to serum levels found in patients treated with this drug^32,51–53^.

Previous studies showed that GLP1R agonists are effective in improving obesity-related hepatic dysfunction^54–56^ but presumably through indirect effects^57^. Studying the specific role of the WAT-liver axis in the semaglutide response using mouse models has been difficult due to the strong effects on feeding behavior and body weight^58^ and because GLP1R expression has been found in various cell types, including macrophages^59,60^ and adipocytes^40,41,61^. In our human iPSC-based system iADIPOs, but not iHEPs or iMACs, responded to semaglutide treatment in monoculture (Supplementary Fig. 4). In accordance, we found little *GLP1R* gene expression in iHEPs and primary human hepatocytes, which confirms previous findings made in whole liver^62,63^. Thus, our multi-tissue model enabled us to investigate the adipocyte-specific effects of semaglutide on inflammation-driven MASLD.

Although semaglutide appears to only target adipocytes directly, it had profound effects on WAT inflammation and HIR. These effects could be due to altered adipocyte-macrophage interactions dampening secretion of proinflammatory cytokines, such as TNFα, from either macrophages and/or adipocytes, similar to what was reported in a mouse model of T2DM^59^. While semaglutide induced lipolysis in WAT, resulting in increased circulating fatty acids, the interconnected iHEP-MPS showed reduced steatosis and improved insulin sensitivity, which we attribute, at least in part, to the insulin-sensitizing effects of adiponectin^64^. Adiponectin action may also explain our finding of better effectiveness of rosiglitazone compared to metformin. Gene editing or specific inhibitors could be applied to our system to define the effects of adipokines such as adiponectin, leptin, resistin and omentin or metabolic and inflammatory pathways that impact hepatic insulin response in the setting of obesity and WAT inflammation^2^. Our findings of decreased inflammation, decreased lipogenesis and increased β-oxidation in the iHEPs of the semaglutide-treated M1-iADIPO-iHEP-MPS align with clinical observations and data from mice, validating the authenticity of our model^56,58,59,65^.

By generating different cell types from the same human iPSC line, our approach reduces genetic biases such as MASLD risk factors, many of which remain to be discovered^66^. However, care needs to be taken to identify and eliminate mechanistically relevant deficiencies in cell maturity and subsequent function of human iPSC-derived cells, including the use of optimized protocols for directed differentiation and in-depth characterization by comparison to primary cells, as we did for iHEPs, iADIPOs and iMACs^8,34–36^. Because our protocols work with other human iPSC lines, the impact of sex and ethnic background on the WAT-liver axis in health and disease could be readily investigated. Moreover, recent advances in directed differentiation of other liver cell types support the feasibility of generating a more complex liver MPS that would facilitate dissecting the role of liver cell interactions in the progression of MASLD, including feedback signaling to the adipose tissue. Along these lines, different types of adipose tissue could be modeled by further developing human iPSC differentiation protocols^67^.

In summary, our human iPSC-based multi-tissue MPS faithfully recapitulates the WAT-liver axis allowing for modeling of obesity-induced disorders and analysis of tissue-specific effects of obesity-targeting drugs. Our model provides an authentic and scalable in vitro approach to advancing the understanding and therapy of obesity-induced hepatic disease and sets the stage for the next generation of isogenic multi-organ disease models.

## Methods

### Ethical statement

This research complies with all relevant ethical regulations. Committee on Laboratory and Environmental Biosafety (CLEB) and Stem Cell Research Oversight (SCRO) Office at University of California Berkeley and University of California San Francisco approved protocols in this study annually. The donor of primary human adipose tissue is part of the Inflammation, Diabetes, Ethnicity and Obesity (IDEO) cohort. All human research participants provided informed consent to take part in the study, including consent for the sharing of potentially identifying information, which was approved by the University of California San Francisco Committee on Human Research (IRB number: 14-14248).

### MPS device fabrication

MPS devices were fabricated in accordance with our previous protocol^35^. Briefly, patterned master templates were fabricated with a thickness of 60 μm by standard photolithography using SU-8 (#3100, MicroChem Corp). The cell chambers were circular with diameter of 1500 μm to provide better adipogenesis support. The microfluidic patterns were replica-molded from the master templates to polydimethylsiloxane (PDMS, Sylgard 184, #NC9285739, FisherScientific) slabs by soft lithography. The inlet/outlets were holed on the media-channel slab using a 0.75 mm biopsy punch (#504529, World Precision Instruments LLC). A polyethylene terephthalate (PET) isoporous membrane (#030060, TRAKETCH, SABEU GmbH & Co. KG) was activated by oxygen plasma (Plasma Equipment Technical Services) at 60 W under ~0.6 Torr for 60 s and then chemically decorated in 2% bis(3-(trimethoxysilyl)propyl)anime solution (#413356, Sigma-Aldrich) in 97% isopropyl alcohol (#A451-4, FisherScientific) and 1% deionized water (Arium Mini, Sartorius) for 30 min at 80°C. After decoration, the membrane was rinsed in pure isopropyl alcohol and stored in anhydrous ethanol solution (#AC611050040, FisherScientific) until further use. For MPS assembly, the PDMS slabs of the cell chamber and media channel were activated by oxygen plasma at 60 W under ~0.6 Torr for 30 s and immediately sandwiched with a size-trimmed decorated PET membrane. To collect cell pellets out of the device, the cell chamber slab was activated for 60 s, resulting ~50% higher bonding strength to the membrane than that of 30 s-treated media channel slab. The device was then baked at 110°C for 30 min for ethanol removal, bonding stabilization, and device sterilization.

### iPSC culture and differentiation

All experiments used the normal human male GM25256 iPSC line (WTC, hPSCreg: UCSFi001-A, Gladstone Institutes, distributed by Coriell Institutes)^68^. All iPSCs were used at passage numbers ranging from 40 to 80. When iPSC colonies reached 80% confluence, they were dissociated using ReLeSR (#100-0483, Stemcell Technologies) and subcultured at a 1:10 ratio in mTeSR Plus medium (#100-0276, Stemcell Technologies) on Matrigel-coated substrates (#356231, Corning). Authentication was confirmed prior to experimentation by SNP analysis. Mycoplasma testing was conducted before all experiments and annually. Sterility was checked daily. The undifferentiated state, characterized by colony morphology, was verified before each differentiation. Pluripotency was assessed before all experiments and regularly, based on signature gene expression comparisons to endodermal and mesodermal differentiated states, and statistically analyzed using *t*-tests.

iMACs were generated as previously described^36^. Briefly, undifferentiated iPSCs were directed to hematopoietic stem cells using STEMdiff Hematopoietic Kit (#05310, Stemcell Technologies). Floating HSCs were collected, then magnetically sorted using anti-CD45 coated beads (#130-045-801, Miltenyi Biotec), and further differentiated to macrophages in ImmunoCult-SF Macrophage Media (#10961, Stemcell Technologies) and 100 ng/mL macrophage-colony stimulating factors (M-CSF; #300-25, Peprotech). Unpolarized M0-iMACs were polarized to M1-iMACs using culture media supplemented with 10 ng/mL LPS (E. coli O111:B4; #LPS25, Millipore) and 20 ng/mL IFNγ (#300-02, Peprotech) for 24 h before use in subsequent experiments. Cells were washed and medium was changed to remove the LPS prior to use in experiments.

Differentiation of iADIPOs were described in our previous study^35^. Briefly, all iPSCs were firstly differentiated into mesenchymal progenitors (iPSC-MSCs) (STEMdiff Mesenchymal Progenitor Kit, #05240, Stemcell Technologies) and then transduced for chemically inducible PPARγ. After 48 h of post-confluent culture, differentiation of iPSC-MSCs was induced in complete media (DMEM/F12 (#11320033, Gibco) containing 1% HEPES (#15630080, Gibco), 1% penicillin/streptomycin (#15140122, Gibco) and 10% fetal bovine serum (#EF-0500-A, Equafetal)) with supplements of 0.25 μM dexamethasone (#D1756, Sigma-Aldrich), 0.25 mM 3-isobutyl-1-methylxanthine (#I5879, Sigma-Aldrich), 100 nM rosiglitazone (#R2408, Sigma-Aldrich) and 500 nM insulin (#0002-8315-01, Humulin R, Eli Lilly) for 4 days (Day 0–3). Subsequent differentiation was completed in media supplemented with insulin and rosiglitazone at the same concentration (Day 4 – 14). Exogeneous PPARγ was induced by 1 μg/mL doxycycline (#D5207, Sigma-Aldrich) from Day 0 until the end. On Day 14, the iADIPOs expressed hallmark genes comparable to primary adipocytes collected from human biopsies^35^.

iHEPs were generated as previously described^34^. Differentiation was performed at 37 °C in 5% CO_2_ and 5% O_2_ unless stated otherwise. Endoderm was induced using endoderm-induction media (EIM), consisting of RPMI 1640 (#11875093, Gibco) containing 2% Gem21 without insulin (#400-962-010, GeminiBio), 1% Glutamax (#35050061, Gibco), 1% non-essential amino acids solution (#11140050, NEAA; Gibco), 0.5 mM sodium butyrate (#B5887, Sigma-Aldrich) and 100 ng/ml activin A (#78001, StemCell Technologies), for 7 days in 20% O_2_. The following compounds were added to EIM during the first 3 days: 3 μM CHIR99201 (#100-1042, StemCell Technologies) on day 1, 20 ng/ml basic fibroblast growth factor (FGFb; #100-18B, Peprotech) and 10 ng/ml bone morphogenetic protein 4 (BMP4; # 120-05ET, Peprotech) on days 1 and 2, 50 nM PI103 (#501932056, Thermo Fisher Scientific) on days 1 to 3, knockout serum replacement (KSR; # 10828010, Gibco) at 2% on day 1, 1% on day 2 and 0.2% on day 3. On days 8 to 17, cells were cultured in hepatic induction media (HIM), consisting of IMDM (#12440053, Gibco) containing 2% Gem21 without insulin, 1% Glutamax, 1% NEAA, 100 nM dexamethasone (#D4902, Sigma-Aldrich), 100 nM insulin (#800-112-005, GeminiBio) and 0.5 mM 1-thioglycerol (#M1753, Sigma-Aldrich). The following compounds were added to HIM: 10 ng/ml FGFb and 20 ng/ml BMP4 between days 8 and 17, 20 ng/ml hepatocyte growth factor (HGF; #100-39, Peprotech) between days 12 and 17. On day 10, cells were detached using 0.25% trypsin-EDTA (#25200056, Gibco) and split 1:2 into matrigel-coated 12- or 24-well plates. On days 18 – 22, cells were cultured in Hepatocyte Culture Media BulletKit (HCM; #CC-3198, Lonza) without epidermal growth factor, including 20 ng/ml oncostatin M (#300-10, Peprotech) and 20 ng/ml HGF in 20% O_2_. Media was changed daily during differentiation and maintenance.

### Primary cells and tissues

Cryopreserved, plateable primary human hepatocytes (Lot: BMO) were purchased from BioIVT; the cells were isolated from a 45 year-old Caucasian male with BMI of 22.6 and no history of excessive alcohol consumption or smoking. Cryopreserved, human pancreatic islets (Lot: 2201233) were purchased from Celprogen; the cells were isolated from a 55 year-old Caucasian male with no history of excessive alcohol consumption or smoking. Primary white adipose tissue, which was used as the standard in our previous study^35^, was biopsied from the subcutaneous fat of a 47 year-old Caucasian female with BMI of 25.6; fasting blood glucose of 94 mg/dL, A1C of 4.8%; exclusion factors include smoking, unstable weight within the last 3 months (>3% weight gain or loss), a diagnosed inflammatory or infectious disease, liver failure, renal dysfunction, cancer, and reported alcohol consumption of >20 grams per day.

### RNA isolation from MPS and gene expression analysis

Cells were collected by first cutting the sandwiched area off the device. Due to unequal plasma activation on the surfaces of media channel and cell chamber slabs, preferable debonding on the interface between media channel slab and the sandwiched membrane was easily achievable by exfoliation. The membrane was sequentially exfoliated off the cell chamber slab to allow direct exposure of the cell pellet. The entire cell chamber slab was then immersed in Trizol (#15596026, Invitrogen) lysis solution with active pipetting to allow efficient lysis of the cell pellet. RNA isolation (#T2010S, NEB), synthesis of cDNA via qRT-PCR (Maxima First Strand cDNA Synthesis Kit for RT-qPCR, # K1641, Thermo Fisher), and qPCR (TaqMan™ Universal PCR Master Mix, # 4304437, Thermo Fisher) in QuantStudio5 (Applied Biosystems, Thermo Fisher) were done according to the manufacturer’s instructions. Oligonucleotide primers for each target gene were pre-designed and synthesized by Integrated DNA Technologies. Relative mRNA expression was determined by the delta-delta-Ct method normalized to *PPIA* or *RPLP0*. Primers for qRT-PCR are listed in Supplementary Table 1. Supplementary Table 2 shows examples of extracted RNA concentrations and efficiency. All genomic analyses were done within 2 weeks of collection.

### Circulating media analysis

Cytokines in circulating media were analyzed by TNFα ELISA (KHC3011), adiponectin ELISA (KHP0041, both from Thermo Fisher) and sent to Eve Technologies (Canada) for analysis with Human Cytokine/Chemokines 65-Plex panel (HD65). Additionally, cytokine concentrations were measured in co-culture supernatants using LEGENDplex Human Inflammation Panel 1 (#740809, Biolegend) according to the manufacturer’s instructions, analyzed using a LSRFortessa flow cytometer and quantified using LEGENDplex software (Biolegend). LDH in media was measured using LDH Cytotoxicity Assay (#8078, ScienCell) according to the manufacturer’s instructions. Non-esterified fatty acids (NEFA) were measured using enzymatic colorimetric assay (NEFA-HR(2) kit including reagents of # 434-91795 and # 436-91995, and standard #270-77000, WAKO) according to the manufacturer’s instructions. Fluorescent fatty acids were quantified by measuring fluorescent intensity (EX/EM 480/520 nm) of the circulating media. Albumin in media was measured using the Human Albumin ELISA Kit (#E88-129, Fortis Life Sciences) according to the manufacturer’s instructions. Urea in media was measured using the QuantiChrom Urea Assay Kit (#DIUR-100, BioAssay Systems) according to the manufacturer’s instructions.

### MPS culture

To ensure the adipogenesis of loaded adipocyte progenitor cells, the initial stage of differentiation was pre-induced in a tissue culture flask. Differentiating iPSC-MSCs on day 4, which contain tiny lipid droplets in > 80% cells, were dissociated (TrypLE Express, Gibco) and centrifuged as cell pellets. Prior to cell loading, 10 wt% MMP cleavable peptides (CQPQGLAKC, GenScript) were dissolved in triethanolamine-buffer (TEOA; 0.3 M, pH 8, #T0449, Sigma-Aldrich) and directly added to the cell pellet with dissolved 3% adhesion side-chain conjugated collagen I short peptide sequence C1^69^ (CGGGF(HYP)GER, GenScript) under a peptide-to-precursor volume ratio of 1:10. The hydrogel-cell slurry with density of 8 × 10^7^ cells/mL was injected into the cell chamber and maintained for 30 min at room temperature to remove uneven loading stress. The cell number in MPS hereafter is estimated based on cell density and loading volume. The MPS was incubated at 37°C for 30 min for crosslinking and then connected with catheter couplers (#SC20/15 and #SP20/12, Instech Laboratories) and tubes (#06422-00, Cole-Parmer). Culture media was perfused at a flow rate of 10 μL/h using a syringe pump (#703007, Harvard Apparatus). After Day 14, freely floating cells were flushed out by a continuous media flow at rate of 30 μL/min. Subsequently, macrophages were loaded at density of 5 × 10^6^ cells/mL into the cell chamber at flow rate of 5 μL/min to yield the ratio of iMACs:iADIPOs at 1:10, matching the physiological ratios in obese WAT^2,70^. At Day 22, fully differentiated iHEPs were loaded into the MPS and cultured for 1 day to allow tissue formation. iMACs and iADIPOs were co-cultured in iADIPO media, including 50 ng/ml M-CSF on Day 14. After 1-day coculture with iMACs, the iMAC-iADIPO-MPS was interconnected with iHEP-MPS at cell ratio of 5:1 (Figs. 2–4). The interconnected iMAC-iADIPO-iHEP-MPS was cultured in 0.2 ml HCM (without insulin and dexamethasone) supplemented with 50 ng/mL M-CSF and 10 ng/mL HGF and cultured for 48 h at 20 μL/h.

### Drug administration

For drug testing, 10 mM metformin (#PHR1084, Sigma-Aldrich), 1 μM rosiglitazone, 0.5 μM dexamethasone, or 1 μM semaglutide (#S9697, Selleckchem) was added to the 0.2 mL circulating media at the beginning of MPS interconnection. To test the tissue-specific effect of GLP1R activation in the M1-iADIPO-iHEP-MPS, 1 μM semaglutide was added to the medium after leaving the M1-iADIPO-MPS or before entering the M1-iADIPO-MPS. The GLP1R antagonists exendin 9–39 (E7269, Sigma) and compound 5D (HY-101116, MedChemExpress) were added to the medium after leaving the M1-iADIPO-MPS at 1 μM each.

### Absorption of fluorescent fatty acids and drugs by PDMS

BSA-complexed fluorescent fatty acids, metformin, rosiglitazone, dexamethasone and semaglutide were diluted in 200 μL deionized water and circulated for 6 or 2 days through a set of interconnected iADIPO-MPS and iHEP-MPS at a flow rate of 20 μL/h. Fatty acid concentration was measured by fluorescence at excitation/emission 480/520 nm and drug concentrations were measured by UV/Vis spectrometry at 240 nm using a monochromator-based microplate reader (SpectraMax i3x, Molecular Devices).

### Functional assays for hormone-stimulated iADIPOs and iHEPs

For dynamic fatty acid assay, adipocytes were preloaded with 2 μM fluorescent fatty acid (Bodipy 500/510 C1, C12, #D3823, Thermo Fisher) in iADIPO media for 48 h and rinsed by iADIPO media without fluorescent fatty acid for 1 h before interconnection to avoid fluorescent carry-over. Fatty acids were quantified based on the relative fluorescence unit (RFU) of the respective MPS. Lipolysis in iADIPOs was induced by 1 μM isoproterenol (#I6504, Sigma-Aldrich) or 1 μM isoproterenol plus 1 μM insulin (#I2643, Sigma-Aldrich). For adipogenic glucose uptake, iADIPO-MPS were infused with low glucose DMEM (1 g/L, #11885084, Gibco) at 37 °C for 3 h for basal uptake, then with 1 μM insulin stimulation for additional 3 h for insulin-stimulated uptake. For hepatic glucose production, iHEP-MPS were infused with low glucose DMEM with 2 mM sodium pyruvate (#11360070, Gibco), 10 mM sodium lactate (#L7022, Sigma-Aldrich) including 100 nm glucagon (#05-23-2700, Sigma-Aldrich) or 100 nm insulin for 1 h. Glucose was measured using the Amplex Red Glucose/Glucose Oxidase Assay kit (#A22189, Thermo Fisher) according to the manufacturer’s instructions.

### Immunofluorescence, lipid staining and image acquisition

MPS were fixed using 4% paraformaldehyde (#15710, Electron Microscopy Sciences) for 2 h. Nuclei and F-actin were stained overnight in PBS including 1% BSA with 300 nM DAPI (#D1306, Thermo Fisher) or 1 unit of phalloidin-iFluor 647 reagent (#ab176759, Abcam), respectively. Samples were mounted using Diamond Antifade (#P36965, Invitrogen) after staining and rinsing. Preloaded fluorescent fatty acids were used to track lipid droplet formation in iADIPO-MPS and iHEP-MPS. Brightfield and fluorescent images were captured by a wide-field fluorescence microscope (AxioObserver Z1, Zeiss) and confocal fluorescence microscopes (LSM710, LSM880, Zeiss). To enable fluorescent comparison, all staining and imaging procedures were kept consistent. Zen (Zeiss) and ImageJ (no plug-ins) were used to analyze fluorescent intensity with consistent settings. Absorbance and fluorescence of assay solutions and circulating media were analyzed using a microplate reader (SpectraMax i3x, Molecular Devices).

### Statistics and reproducibility

All results were statistically analyzed using JMP 11 (SAS Institute) or Prism (GraphPad). Paired two-tailed *t*-test with assumption of equal variance was used to compare the difference between two measures from the same MPS. Unpaired two-tailed *t*-test with the same assumption was used for results from different MPS. One-way analysis of variance (ANOVA) for 3 or more samples in one group and two-way ANOVA for multiple groups with 3 or more samples each group, and then post-hoc test for multiple comparisons with corrections were performed to determine significant pair(s): Tukey’s test was used in most analyses except in Fig. 1j where Dunnett’s test was used to determine the significance between iADIPO:iHEP ratios and iADIPO control. Detailed test methods, *p*-values, degrees of freedom, confidence intervals and exact sample size for all results are listed in Supplementary Data 1. *p* < 0.05 was considered significant (*), *p* < 0.005 highly significant (**). Results are shown as mean values with error bars representing standard deviation (SD). All experiments were repeated independently (biological replicates, n) with similar results at least 3 times (except Supplementary Fig. 5b).

### Reporting summary

Further information on research design is available in the Nature Portfolio Reporting Summary linked to this article.

## Supplementary information

Supplementary Information

Peer Review File

Description of Additional Supplementary Files

Supplementary Data 1

Reporting Summary

## Source data

Source Data

## Data Availability

Primer sequences, sample sizes, statistic methods and *p*-values are provided in Supplementary Tables 1 and 2, and Supplementary Data 1. Source data are provided with this paper.
