# Supplementary material for: Adipocyte inflammation is the primary driver of hepatic insulin resistance in a human iPSC-based microphysiological system
Source: Nat Commun. 2024 Sep 12;15:7991. doi: 10.1038/s41467-024-52258-w (PMC11393072; doi:10.1038/s41467-024-52258-w)
Supplement: Supplementary file 1 — Supplementary Information [file 41467_2024_52258_MOESM1_ESM.pdf]

Supplementary Information

Adipocyte inflammation is the primary driver of hepatic insulin resistance in a human iPSC-based microphysiological system

Lin Qi[+,1], Marko Groeger[+,2], Aditi Sharma[3], Ishan Goswami[4], Erzhen Chen[1], Fenmiao Zhong[1], Apsara Ram[3], Kevin Healy[4,5], Edward C. Hsiao[3], Holger Willenbring*[,2], Andreas Stahl*[,1]

[+]Authors contributed equally to this paper

[*]Correspondence to: Holger Willenbring (holger.willenbring@ucsf.edu) and Andreas Stahl (astahl@berkeley.edu)

Table of Content:

Supplementary Figure 1. iADIPO-iHEP transwell co-culture.

Supplementary Figure 2. PDMS absorption in cell-free MPS.

Supplementary Figure 3. Specific effects of iMACs and iADIPOs on iHEP-MPS.

Supplementary Figure 4. Effects of semaglutide treatment on individual cell types.

Supplementary Figure 5. *GLP1R* expression in iPSC-derived and primary human cells.

Supplementary Table 1. Primers for qRT-PCR.

Supplementary Table 2. RNA yield from 1 well of a 6-well tissue culture plate and 1 MPS.

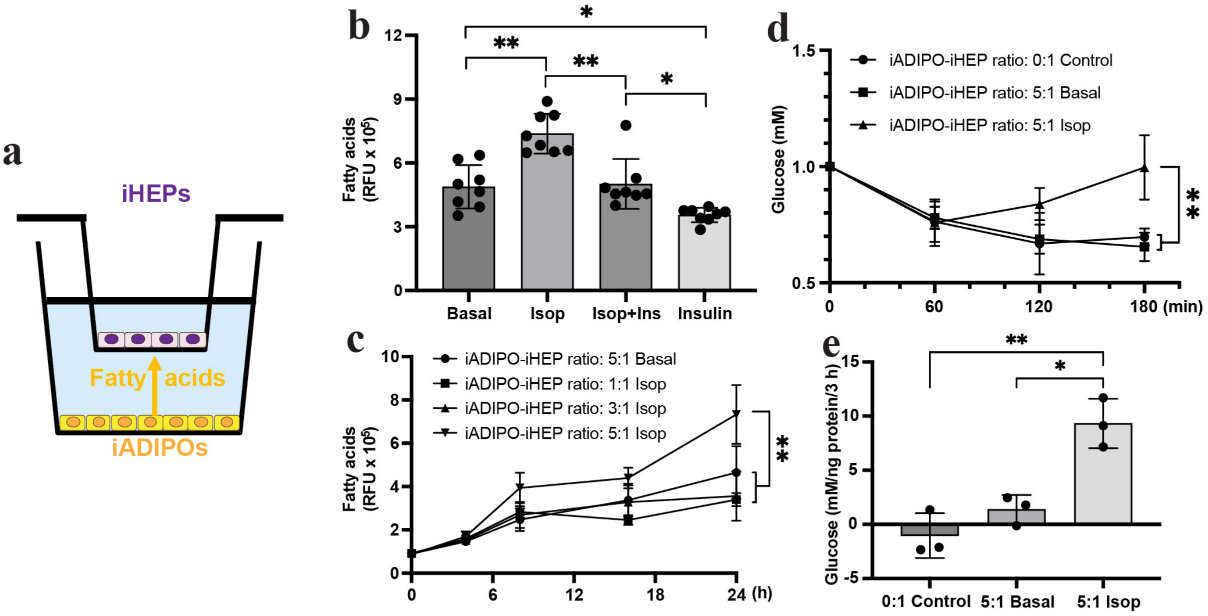

Supplementary Figure 1. iADIPO-iHEP transwell co-culture. (a) Schematic of transwell co-culture of iHEPs and iADIPOs. (b) Isoproterenol (Isop)-induced release of fatty acids into media by iADIPOs with and without insulin stimulation. n=8 referring to media. (c) Time course of fatty acid uptake into iHEPs co-cultured with iADIPOs. n=3 referring to iHEPs. (d) Time course of glucose release into media of insulin-stimulated iHEPs after co-culture with iADIPOs. Insulin was added at the beginning (0 minutes, min). n=4 referring to media. (e) Normalized glucose release into media by iHEPs after 3 hours (h) of insulin stimulation after co-culture with iADIPOs. n=3 referring to media. All data are mean ± SD. p-values were calculated by one-way ANOVA followed by Tukey's test in (b,e) and two-way ANOVA then Tukey's test in (c,d). $^*p<0.05$; $^{**}p<0.005$. Statistical methods and exact p-values are shown in Supplementary Data 1. Source data are provided as Source Data file.

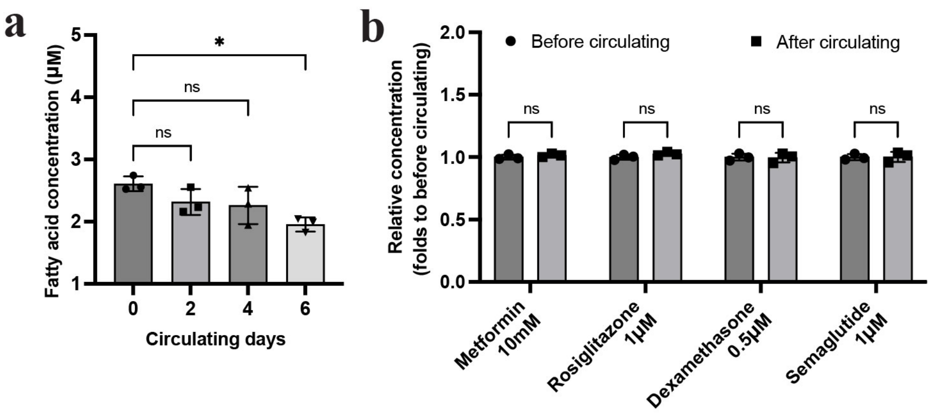

Supplementary Figure 2. PDMS absorption in cell-free MPS. (a) Fluorescent fatty acid concentrations in media circulating for up to 6 days. n=3 referring to media. p-values were determined by one-way ANOVA followed by Dunnett's test by comparing circulating days to day 0. (b) Relative drug concentrations in media before and after circulation for 48 hours. n=3 referring to media. p-values were determined by unpaired two-tailed t-tests. *p<0.05; **p<0.005; ns, p>0.05. All data are mean ± SD. Statistical methods and exact p-values are shown in Supplementary Data 1. Source data are provided as Source Data file.

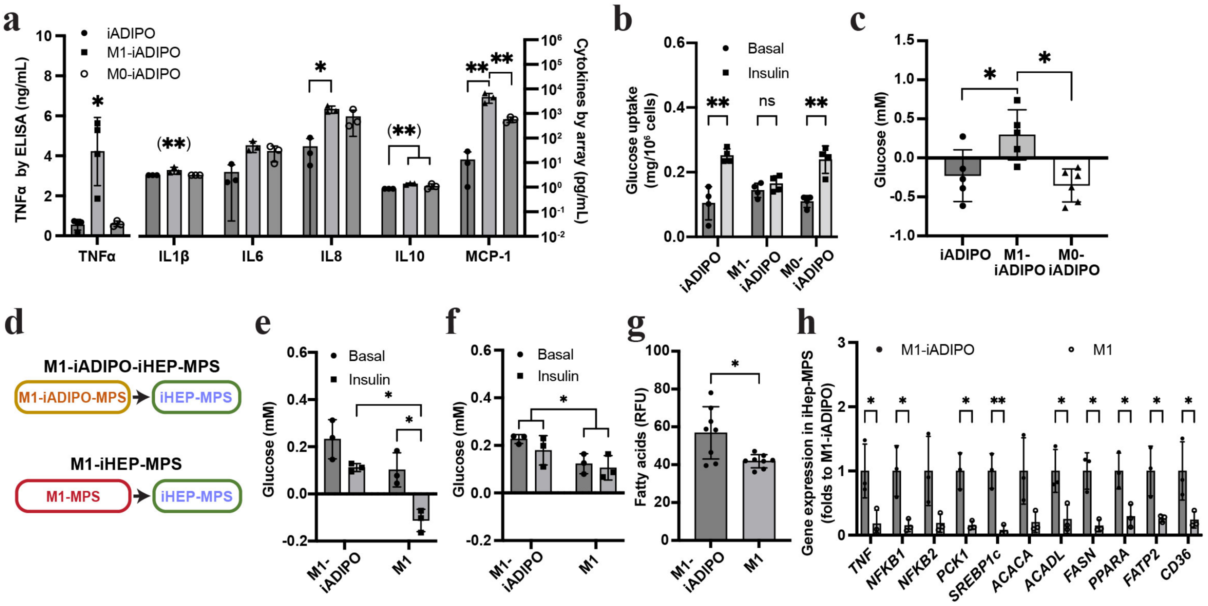

Supplementary Figure 3. Specific effects of iMACs and iADIPOs on glucose and lipid metabolisms in iHEP-MPS. (a) Cytokine levels in media circulating between iHEP-MPS and iADIPO-MPS without iMACs, with M1-iMACs or with M0-iMACs 48 hours after interconnection. n=3 except n=4 for iADIPO and M1-iADIPO TNFα. n referring to media. (**) indicate values that were set to detection limit of the assay. (b) Glucose uptake in iADIPO-MPS cultured without iMACs, with M1-iMACs or with M0-iMACs for 48 hours. n=4 referring to media. (c) Glucose levels in media of insulin-stimulated iHEP-MPS after interconnection with iADIPO-MPS without iMACs, with M1-iMACs or with M0-iMACs for 48 hours. n=5 except n=6 for M0-iADIPO. n referring to media. (d) Schematic of interconnected MPS conditions with (top) or without (bottom) iADIPOs. (e,f) Glucose levels in circulating media 24 hours (e) and 48 hours (f) after interconnection. n=3 referring to media. (g,h) Basal lipid uptake (g) (n=8 referring to MPS) and gene expression (h) (n=3 referring to MPS) in iHEP-MPS after 48 hours of interconnection. p-values were determined by paired two-tailed t-test in (b,e,f) in Basal vs Insulin comparison, one-way ANOVA followed by Tukey's test in comparison of three conditions in (a,c) and two-way ANOVA then Tukey's test in cross-group comparison in (e), and unpaired two-tailed t-test in (g,h). The significance of iADIPO effectiveness in (f) was determined by ANOVA without post-hoc test. All data are mean ± SD. *p<0.05; **p<0.005; ns, p>0.05. Statistical methods and exact p-values are shown in Supplementary Data 1. Source data are provided as Source Data file.

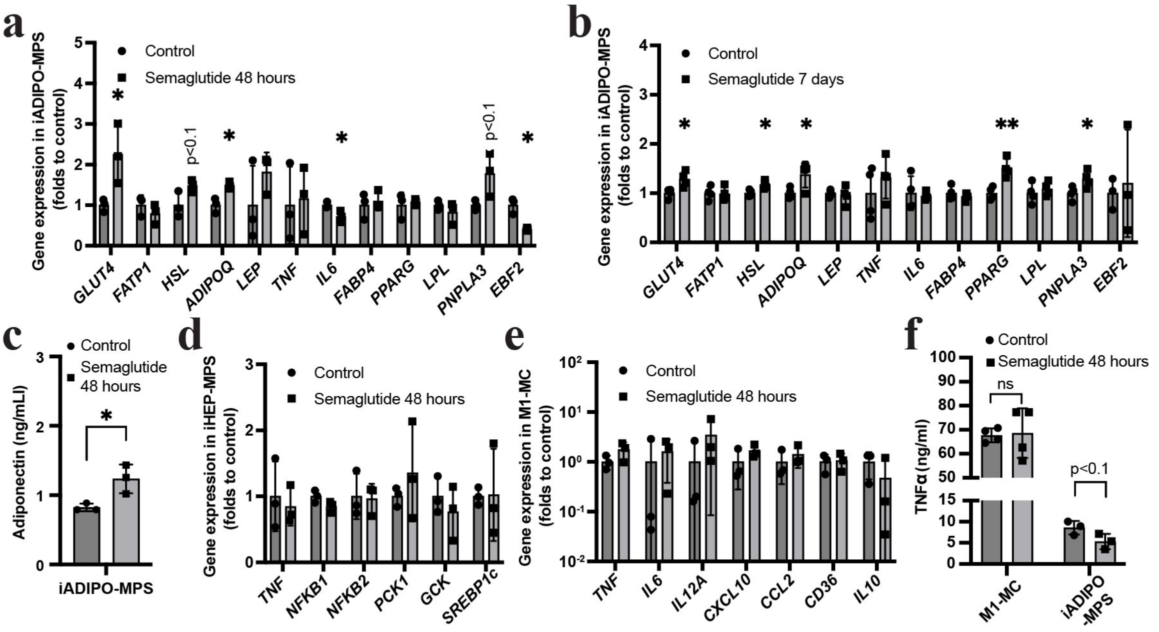

Supplementary Figure 4. Effects of semaglutide treatment on individual cell types. (a,b) Gene expression in iADIPO-MPS after 48 hours (a) (n=3 referring to MPS) and 7 days (b) (n=4 except n=3 for *EBF2* referring to MPS). (c) Adiponectin levels in circulating media. n=3 referring to media. (d,e) Gene expression in iHEP-MPS (d) (n=3 referring to MPS) and M1-iMACs in monoculture (MC; e) (n=3 referring to iMACs). (f) TNFα levels in circulating media. n=4 in M1-MC referring to wells and n=3 in iADIPO-MPS referring to MPS. p-values were determined by unpaired two-tailed t-test. *p<0.05; **p<0.005; ns, p>0.05. All data are mean ± SD. Statistical methods and exact p-values are shown in Supplementary Data 1. Source data are provided as Source Data file.

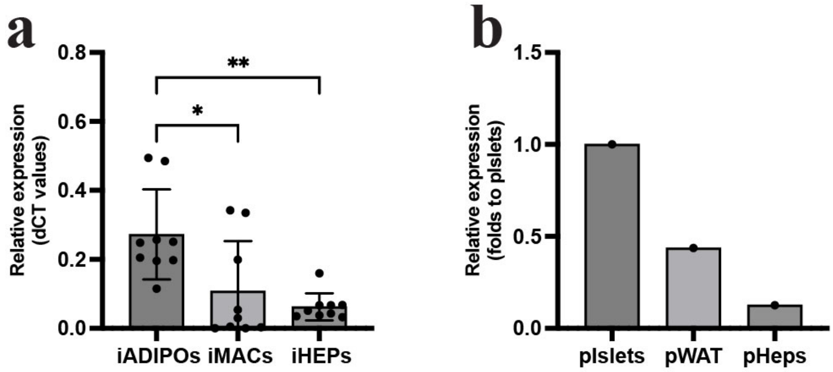

Supplementary Figure 5. *GLP1R* expression in iPSC-derived and primary human cells. (a) *GLP1R* gene expression in iPSC-derived cells. n=9 referring to MPS for iADIPOs and iHEPs, and to wells for iMACs. *p<0.05; **p<0.005. p-values were determined by one-way ANOVA followed by Tukey's test. (b) *GLP1R* gene expression in primary human pancreatic islets (pIslets), primary human WAT (pWAT) and primary human hepatocytes (pHeps). pWAT contains adipocytes, tissue-resident macrophages and other cell types. n=1 referring to cells/tissue. All data are mean ± SD. Statistical methods and exact p-values are shown in Supplementary Data 1. Source data are provided as Source Data file.

Supplementary Table 1. Primers for qRT-PCR.

Gene	Direction	Sequence	Gene	Direction	Sequence
TNF	Forward	CCTCTCTCTAATCAGCCCTCTG	*GLUT4 (SLC2A4)*	Forward	TCCAACAGAT AGGCTCCGAA
	Reserve	GAGGACCTGGGAGTAGATGAG		Reserve	CCCAATGTTGTACCCAAACTG
NFKB1	Forward	CAGGAGACGTGAAGATGCTG	*FATP1 (SLC27A)*	Forward	AGTGTCTCATCTATGGGCTGA
	Reserve	AGTTGAGAATGAAGGTGGATGA		Reserve	AGGTAGCGGCAGATCTCC
NFKB2	Forward	ATGGAGAGTTGCTACAACCCA	*HSL (LIPE)*	Forward	AGTATGTCACGCTGCATAAGG
	Reserve	CTGTTCCACGATCACCAGGTA		Reserve	GAAGGACACCAGCCCAAT
PCK1	Forward	TCATTTAAGGGCCATCAACC	*ADIPOQ*	Forward	CCTCACTTCCATTCTGACTGC
	Reserve	CTCATCAATGCCTTCCCAGT		Reserve	AGCATCCTGAGCCCTGAT
G6PC	Forward	ACTGGCTCAACCTCGTCTTTA	*LEP*	Forward	TGGCTTCCAGGTATCTCCA
	Reserve	CGGAAGTGTTGCTGTAGTAGTCA		Reserve	AGAGTGGCTTAGAGGAGTCAG
GCK	Forward	CCGCAAGCAGATCTACAACA	*IL6*	Forward	GCAGATGAGTACAAAAGTCCTGA
	Reserve	AGCTTGTACACGGAGCCATC		Reserve	TTCTGTGCCTGCAGCTTC
SREBP1c	Forward	CCATGGATTGCACTTTCGAA	*IL10*	Forward	GCGCTGTCATCGATTTCTTC
	Reserve	GGCCAGGGAAGTCACTGTCTT		Reserve	TCACTCATGGCTTTGTAGATGC
ACACA	Forward	CTGCCCACATCTCATCCAAA	*IL12A*	Forward	CAGTTATTGATGAGCTGATGCAG
	Reserve	GTACATCGCTGACACTAGCTAC		Reserve	CATGAAGAAGTATGCAGAGCTTG
ACACB	Forward	GCCTATGAGATGTTCCGCAA	*CCL2*	Forward	AGCAGCCACCTTCA TTCC
	Reserve	CGTAATGATCCGCCATCTTGA		Reserve	GCCTCTGCACTGAGATCTTC
ACADL	Forward	GCAAT AGCAATGACAGAGCCT	*CXCL10*	Forward	GACATATTCTGAGCCTACAGCA
	Reserve	GCAACT ACAATCACAACATCACT		Reserve	CAGTTCT AGAGAGAGGT ACTCCT
FASN	Forward	TTTGATGCCTCCTTCTTCGG	*CD36*	Forward	TCAATTCGTCTAATCATTGGAAA GC
	Reserve	CGGAGTGAATCTGGGTTGATG		Reserve	CCTGTTTACTTTCTGCATCTGC
CYP2E1	Forward	AGCCAGAACACTTCCTGAATG	*PPARG*	Forward	GTTTCAGAAATGCCTTGCAGT
	Reserve	GCACACAACAAAAGAAACAACTC		Reserve	GGATTCAGCTGGTCGATATCAC
PPARA	Forward	TGGCAAGACAAGCTCAGAAC	*FABP4*	Forward	ACTTGTCTCCAGTGAAAACTTTG
	Reserve	CGTCCTCACATGACACTGAATC		Reserve	ATCACATCCCCATTCACACT
GLP1R	Forward	TAACCTCAGCCAAACACAGA	*LPL*	Forward	AGTCAGAGCCAAAAGAAGCAG
	Reserve	AAGCAACCCAGACACACAA		Reserve	TTGGTATGGGTTTCACTCTCAG
PPIA	Forward	GTGGCGGATTTGATCATTTGG	*PNPLA3*	Forward	CCTTGGTATGTTCCTGCTTCA
	Reserve	CAAGACTGAGATGCACAAGTG		Reserve	GTTGTCACTCACTCCTCCATC
RPLP0	Forward	GCAGATCCGCATGTCCCTT	*EBF2*	Forward	CTATGTCAGGCTCATCGACTC
	Reserve	TGTTTTCCAGGTGCCCTCG		Reserve	CAGCATCGACT ACACATCACT

Supplementary Table 2. RNA yield from 1 well of a 6-well tissue culture plate and 1 MPS. Cells from 1 well of a 6-well plate can be loaded into approximately 4 MPS (ratio 1:4). Measurements are based on iHEPs.

RNA yield (µg/mL)	Isolated from	
Sample #	1 well	1 MPS
1	191	19.6
2	269	27.8
3	471	54.5
4	393	51.7
5	336	49.7
6	269	49.1
7	234	53
8	270	27.2
Average	304.125	41.575
Loading ratio	1	4
Total RNA	304.125	166.3
Net yield	N/A	54.68%
