# Supplementary material for: Adipocyte inflammation is the primary driver of hepatic insulin resistance in a human iPSC-based microphysiological system
Source: Nat Commun. 2024 Sep 12;15:7991. doi: 10.1038/s41467-024-52258-w (PMC11393072; doi:10.1038/s41467-024-52258-w)
Supplement: Supplementary file 3 — Description of Additional Supplementary Files [file 41467_2024_52258_MOESM3_ESM.pdf]

<u>Description of Additional Supplementary File</u>

Supplementary Data 1: Exact sample numbers (n) and detailed statistical analyses of all data, including test methods, p-values, two-side or one-side, paired or unpaired, correction methods in multiple comparison, degree of freedom, confidence intervals.
