# Supplementary material for: Adipocyte inflammation is the primary driver of hepatic insulin resistance in a human iPSC-based microphysiological system
Source: Nat Commun. 2024 Sep 12;15:7991. doi: 10.1038/s41467-024-52258-w (PMC11393072; doi:10.1038/s41467-024-52258-w)
Supplement: Supplementary file 2 — Peer Review File [file 41467_2024_52258_MOESM2_ESM.pdf]

nature portfolio

Peer Review File

Adipocyte inflammation is the primary driver of hepatic insulin resistance in a human iPSC-based microphysiological system

Open Access This file is licensed under a Creative Commons Attribution 4.0 International License, which permits use, sharing, adaptation, distribution and reproduction in any medium or format, as long as you give appropriate credit to the original author(s) and the source, provide a link to the Creative Commons license, and indicate if changes were made. In the cases where the authors are anonymous, such as is the case for the reports of anonymous peer reviewers, author attribution should be to 'Anonymous Referee' followed by a clear attribution to the source work. The images or other third party material in this file are included in the article's Creative Commons license, unless indicated otherwise in a credit line to the material. If material is not included in the article's Creative Commons license and your intended use is not permitted by statutory regulation or exceeds the permitted use, you will need to obtain permission directly from the copyright holder. To view a copy of this license, visit http://creativecommons.org/licenses/by/4.0/.

REVIEWER COMMENTS

Reviewer #1 (Remarks to the Author):

• Overall, the manuscript by Qi and Groeger, et al. is impactful and represents a clear step forward in the overall effort to further incorporate the use of all-human MPS as important tools for providing a better mechanistic understanding of MASLD-T2DM disease pathophysiology and as a drug testing platform for this disease. The studies in this manuscript are among the first (if not the first) that couples the use of a white adipose tissue (iADIPO) MPS platform with a liver (iHEP) MPS platform (using isogenic iPSCs) to a) examine the effect of inflammation induced by the addition of pro-inflammatory M1 macrophages to the iADIPO MPS on iHEP MPS functionality and progression of MASLD/T2DM and b) demonstrate the use of this coupled MPS as a drug testing platform to evaluate therapeutics to alleviate critical disease features (hepatic steatosis and HIR) associated with the progression of MASLD (metabolic-dysfunction associated steatotic liver disease) is now the accepted term.

• The critical findings of this manuscript include:
1. Using the coupled iADIPO-iHEP MPS, it is demonstrated that the pre-loading of the iADIPO MPS component with FFAs at various iADIPO:iHEP ratios induces FFA uptake in the iHEP component. However, the critical finding is that evidence of HIR is only observed at non-physiological ratios of iADIPO:iHEP (30:1), suggesting that other factors other than total fat mass are required to drive iHEP dysfuncton(HIR).
2. As an extension of point #1, a key disease-relevant point of this manuscript is that the addition of M1-iMACS (macrophages) to the M1-iADIPO MPS is sufficient to induce steatosis, HIR, and increased expression of pro-inflammatory markers in the iHEP MPS component of the coupled system. Importantly, steatosis and HIR under these conditions are demonstrated under a physiologically relevant ratio of M1-iADIPO-iHEP (5:1) and, in contrast, in coupled MPS supplemented with unpolarized (M0) iMACS, there was no evidence of increased production of pro-inflammatory factors and HIR.
3. To complement the MASLD-T2D disease progression findings highlighted in points #1 and #2 this manuscript also utilizes the coupled iADIPO-iHEP MPS as a drug testing platform to assess the efficacy of several drugs on the MASLD/T2D phenotypes observed in their coupled MPS. To validate the IADIPO-iHEP system as a drug testing platform, it is shown that treatment with the insulin sensitizers rosiglitazone and metformin alleviate M1-iMAC-induced abnormalities in lipid and glucose metabolism in the iADIPO and iHEP MPS. Furthermore, it is also demonstrated that treatment with the GLP1R agonist, semaglutide, reverse both hepatic steatosis and HIR via a reduction in WAT inflammation in the M1-iADIPO MPS, suggesting a disease-relevant mechanistic link between the M1-iADIPO and iHEP MPS.

• Major weaknesses of this manuscript to address include:
1. While it is beneficial that isogenic iPSCs are used in these studies, these iPSCs are derived from

a single male iPS source. Therefore, sex-specific differences using this model cannot be addressed and this limitation should be made clear in the discussion/interpretation of this work.
2. While in lines 63-65 of the Introduction, the drawbacks of MPS constructed with primary cell lines are pointed out (e.g., donor heterogeneity; genetic abnormalities) in comparison to MPS constructed with isogenic iPSCs, it should also be made clear that there is considerable value in using isogenic primary cells in MPS models to study disease progression and response to drug as critical internal benchmarks to models constructed with iPSCs, as functional maturity is always an issue with iPSC-based model systems.
3. Related to comment 2, it would make the functional characterization of iADIPO and iHEP systems much stronger if similar experiments were performed in MPS constructed with primary cells that could serve as a benchmark for the iPSC functional metrics described that are critical for later parts of the paper where the two individual MPS systems are coupled.

4. For the drug testing studies (Figs 3 and 4) there are several issues to address:
a. The description of the drug treatment experimental setup is unclear. It seems like the drugs were added to the circulating media 2 days after the M1-iADIPO and iHEP MPS had been coupled and then treatment lasted for 2 more days. It would be helpful to explain this setup more clearly or create a simple schematic highlighting the critical steps of the drug treatment studies.
b. Both the iADIPO and iHEP MPS are PDMS-based platforms and there was no evaluation of drug loss due to PDMS absorption for any of the drugs tested, therefore we do not know the actual concentration of drug in MPS that resulted in the reversal of MASLD-T2D disease phenotypes. It would be useful to determine drug loss in cell-free MPS for each of these compounds so that functional concentration of drug can be obtained.
c. Related to comments 2 and 3, it would be very useful and supportive to have drug response data from MPS constructed with primary cells to use as a benchmark for the iPSC results.

5. The use of iPSCs in MPS is a stepwise process, and an important limitation of this work is that the iHEP MPS is constructed with only iPSC-derived hepatocytes. Thus, a key point for the authors to discuss is that MASLD disease progression, including the development of steatosis and progression towards HIR involves multiple liver cell types (HSCs, LSECs, KCs), and that their current model may provide even more intricate analysis if these cell types are included in future iterations of the platform. This may be particularly useful in using the iADIPO-iHEP system as a drug development platform for more thoroughly evaluating the effects of GLP1R agonists like semaglutide in a liver model system that incorporates more of the relevant cell types.

• Minor weaknesses of this manuscript to address include:
1. In the Introduction (line 45) and subsequently throughout the rest of the manuscript, it should be noted that recently the terminology for metabolic-dysfunction associated fatty liver disease (MAFLD) has been changed to metabolic-dysfunction associated steatotic liver disease (MASLD) [PMID: 38223415]. It would be useful to change the terminology to reflect the change in nomenclature in this manuscript.
2. In the Introduction (lines 61 and 62), the citations (refs 17 and 18) that reference the use of liver-on-a-chip models do not sufficiently reflect the role that these models have played in studying both MASLD and T2DM. There are multiple commercial and academic groups who have published

MASLD/T2D-specific applications of their liver MPS platforms that should be described here so that the contributions of this manuscript can be put in better perspective.

Reviewer #2 (Remarks to the Author):

In this study, the authors established the unique microphysiological system (MPS) that interconnects iPSC-differentiated isogenic adipocytes and hepatocytes. By co-culturing adipocytes with iPSC-derived macrophages, the authors also mimicked adipose tissue inflammation *in vitro* and reproduced the lipid influx from adipocytes to hepatocytes and hepatic insulin resistance found in fatty liver in patients with obesity and type 2 diabetes. Using this system, the authors tested directly the effect of a couple of anti-diabetic drugs such as metformin and rosiglitazone on lipid accumulation in iPSC-derived hepatocytes, and showed that a GLP-1RA semaglutide ameliorates lipid accumulation and insulin resistance in hepatocytes through the adipose-to-liver interaction. This study should provide clues to understand how the adipose tissue interacts with the liver, where lipid accumulation and insulin resistance occur during the development of obesity-induced adipose tissue inflammation, and even how some of antidiabetic drugs can act directly on the system to prevent and/or treat the diseased states. There are several concerns to be addressed.

Major comments

1. Although this MPS is unique in that the authors could test the direct effect of chemicals/drugs on adipocytes, hepatocytes, and even macrophages within this system. The authors would pursue the mechanism underlying their observations. As the unique simplified *in vitro* model system, the authors would provide how the new findings obtained herein might be extrapolated to the more complex setting; using, for instance, mice with pharmacological intervention.

2. In the previous work, the authors reported that macrophage-derived inflammatory cytokines induce hepatic insulin resistance by inhibiting insulin signaling in iPSC-derived hepatocytes (**Nat Commun.** 2023). Given that macrophages are the upstream of hepatocytes in the new system, macrophages-derived soluble factors may act directly on hepatocytes as well as adipocytes. The authors described transcriptional changes in hepatocytes, focusing on inflammation and glucose/lipid metabolism, which would, however, occur after the addition of soluble factors such as TNFα in hepatocytes. Unbiassed transcriptomic analysis in iHEP-MPS should be important.

3. A recent study reported the marked heterogeneity of adipocytes and macrophages in human adipose tissue, which might affect the adipose tissue phenotype (**Nature** 603:926-933, 2022). Are adipocytes and/or macrophages used relatively homogenous or phenotypically mixed in this system? Single cell/nucleus analysis of adipocytes in this system would be helpful.

4. The authors stated that the GLP-1 analogue had subtle effects on macrophages and hepatocytes,

based on the qPCR analysis of the cell types. Given the expression pattern of GLP-1R, however, I dare say that the GLP-1RA have considerable effect on hepatocytes and macrophages. The authors are required to verify how lipid accumulation in iHEP-MPS changes, when GLP-1 signaling is disrupted in adipocytes (e.g. knockdown of GLP-1R), so that they could suggest that the GLP-1RA acts more effectively on adipocytes than other cell types.

Minor comment

5. The legends for Figure 2g and 2h do not correspond to Figures 2g and 2h.
6. Line 140-141. Lipid accumulation in iHEP should be quantified in Figure 3a.

Reviewer #3 (Remarks to the Author):

This is an interesting article that claims to develop the first human adipose tissue/liver MPS system with the inclusion of inactive and active monocytes. All three cell types have been derived from the same induced pluripotent stem cell line. They indicate that the presence of inflammation from the macrophages in the presence of the adipocytes cause lipid accumulation in the hepatocytes as well as insulin resistance. They monitored the systems response to two known therapeutics as well as a GLP1 receptor agonist that improved hepatocyte function by acting on the adipocytes.

While interesting, this paper has several major flaws that need to be addressed before publishing. First, they claim this is the first multi-organ hepatocyte/adipocyte MPS, which is false, as this was published by Slaughter et al. in Scientific Reports (2021). The authors knew this was false as they then reference this paper a few lines later to claim that it utilized just primary cells and cell lines are not as useful for this type of research, the latter statement also being false in that cell lines were not used in this paper and the primary cells were well-characterized first before being combined into the multi-organ system. In addition, primary cells if characterized properly are excellent models for in vitro experiments and can be useful for capturing population heterogeneity. In addition, most of Figure 2 simply repeats the results from the Slaughter paper and they utilized metformin as one of the drugs that was also in the Scientific Reports paper, which indicated that it was only effective in the MPS model at supraphysiological concentrations and the reason why this drug may not be effective clinically. No mention of this is made in this paper, and as a matter of fact, concentrations for the drugs are not listed in the figures or text and there is no section in the Methods that describes drug administration.

A highlight of this paper is using all three cell types from the same stem cell line, but to some degree, this defeats the argument that primaries are a bad model because of their heterogeneity. Whereas it is possible that their pluripotent stem cells are derived from cells from a high performing outlier and they would not know this unless they did multiple cell lines. The paper does make important points for insulin resistance as well as possible therapeutics that block adipocyte to hepatocyte communication which could be a significant finding.

To be acceptable, the paper would have to correct the untrue statements by properly referencing the original paper and highlighting the differences to establish a strong premise for the work to be published in a high level journal. To also strengthen the premise, they should reference additional publications on multi-organ MPS for other indications to highlight the significance and uniqueness of their approach.

Reviewer #4 (Remarks to the Author):

I co-reviewed this manuscript with one of the reviewers who provided the listed reports. This is part of the Nature Communications initiative to facilitate training in peer review and to provide appropriate recognition for Early Career Researchers who co-review manuscripts.

Point-by-point response to reviewers' comments

Our specific point-by-point reply is as follows:

Reviewer #1 (Remarks to the Author):

Overall, the manuscript by Qi and Groeger, et al. is impactful and represents a clear step forward in the overall effort to further incorporate the use of all-human MPS as important tools for providing a better mechanistic understanding of MASLD-T2DM disease pathophysiology and as a drug testing platform for this disease. The studies in this manuscript are among the first (if not the first) that couples the use of a white adipose tissue (iADIPO) MPS platform with a liver (iHEP) MPS platform (using isogenic iPSCs) to a) examine the effect of inflammation induced by the addition of pro-inflammatory M1 macrophages to the iADIPO MPS on iHEP MPS functionality and progression of MASLD/T2DM and b) demonstrate the use of this coupled MPS as a drug testing platform to evaluate therapeutics to alleviate critical disease features (hepatic steatosis and HIR) associated with the progression of MASLD (metabolic-dysfunction associated steatotic liver disease) is now the accepted term.

The critical findings of this manuscript include:
1. Using the coupled iADIPO-iHEP MPS, it is demonstrated that the pre-loading of the iADIPO MPS component with FFAs at various iADIPO:iHEP ratios induces FFA uptake in the iHEP component. However, the critical finding is that evidence of HIR is only observed at non-physiological ratios of iADIPO:iHEP (30:1), suggesting that other factors other than total fat mass are required to drive iHEP dysfuncton (HIR).
2. As an extension of point #1, a key disease-relevant point of this manuscript is that the addition of M1-iMACS (macrophages) to the M1-iADIPO MPS is sufficient to induce steatosis, HIR, and increased expression of pro-inflammatory markers in the iHEP MPS component of the coupled system. Importantly, steatosis and HIR under these conditions are demonstrated under a physiologically relevant ratio of M1-iADIPO-iHEP (5:1) and, in contrast, in coupled MPS supplemented with unpolarized (M0) iMACS, there was no evidence of increased production of pro-inflammatory factors and HIR.
3. To complement the MASLD-T2D disease progression findings highlighted in points #1 and #2 this manuscript also utilizes the coupled iADIPO-iHEP MPS as a drug testing platform to assess the efficacy of several drugs on the MASLD/T2D phenotypes observed in their coupled MPS. To validate the iADIPO-iHEP system as a drug testing platform, it is shown that treatment with the insulin sensitizers rosiglitazone and metformin alleviate M1-iMAC-induced abnormalities in lipid and glucose metabolism in the iADIPO and iHEP MPS. Furthermore, it is also demonstrated that treatment with the GLP1R agonist, semaglutide, reverse both hepatic steatosis and HIR via a reduction in WAT inflammation in the M1-iADIPO MPS, suggesting a disease-relevant mechanistic link between the M1-iADIPO and iHEP MPS.

We appreciate the reviewer's feedback on the potential of our model for improving the mechanistic understanding of MASLD and T2DM.

Major weaknesses of this manuscript to address include:
1. While it is beneficial that isogenic iPSCs are used in these studies, these iPSCs are derived from a single male iPS source. Therefore, sex-specific differences using this model cannot be addressed and this limitation should be made clear in the discussion/interpretation of this work.

We agree with the reviewer's comment and addressed the sex-specific limitations of our study in the discussion section (lines 302-304) of our revised manuscript.

2. While in lines 63-65 of the Introduction, the drawbacks of MPS constructed with primary cell lines are pointed out (e.g., donor heterogeneity; genetic abnormalities) in comparison to MPS constructed with isogenic iPSCs, it should also be made clear that there is considerable value in using isogenic primary cells in MPS models to study disease progression and response to drug as critical internal benchmarks to models constructed with iPSCs, as functional maturity is always an issue with iPSC-based model systems.

The reviewer's point is well-taken. To provide a more comprehensive overview of available models using primary cells, we expanded the introduction (lines 70-91) and discussion (lines 300-312) sections in our revised manuscript. These new parts also provide a more detailed and nuanced summary of the advantages for MPS development of human iPSC-derived cells over human primary cells. We acknowledge that limitations in cell maturation are a general problem of human iPSC-derived cells and highlight the importance of using state-of-the-art iPSC differentiation protocols and in-depth characterization, including comparison to primary cells, to be confident in the efficacy of iPSC-derived cells as we have done for iMACs, iADIPOs and iHEPs[1–4] (lines 300-312).

3. Related to comment 2, it would make the functional characterization of iADIPO and iHEP systems much stronger if similar experiments were performed in MPS constructed with primary cells that could serve as a benchmark for the iPSC functional metrics described that are critical for later parts of the paper where the two individual MPS systems are coupled.

We agree with the reviewer's point and have validated iMACs, iADIPOs and iHEPs generated with our recently published protocols by in-depth comparison to human primary cells in prior publications[1–4]. We added this information to the discussion section (lines 300-305) of our revised manuscript. Our published findings demonstrate that our iPSC-derived cells perform similar to primary cells, which is validated in our current manuscript. MPS built with these cells show responses to prototypical insulin-sensitizing or anti-inflammatory drugs that are consistent with findings by others made using primary cells or in patients (Figure 3 and lines 258-271)[5–12].

4. For the drug testing studies (Figs 3 and 4) there are several issues to address:
a. The description of the drug treatment experimental setup is unclear. It seems like the drugs were added to the circulating media 2 days after the M1-iADIPO and iHEP MPS had been coupled and then treatment lasted for 2 more days. It would be helpful to explain this setup more clearly or create a simple schematic highlighting the critical steps of the drug treatment studies.

We apologize for the unclear description of the experiments. As part of the revision of our manuscript, we have included schematic illustrations of the experimental setup and timing of drug administration in the MPS in Figures 3a and 4h. We also added a new section, "Drug administration", to the methods section (lines 465-473) that provides detailed information. To clarify the timing of administration, we added all drugs immediately after interconnection of the MPS for a total treatment time of 48 hours.

b. Both the iADIPO and iHEP MPS are PDMS-based platforms and there was no evaluation of drug loss due to PDMS absorption for any of the drugs tested, therefore we do not know the actual concentration of drug in MPS that resulted in the reversal of MASLD-T2D disease phenotypes. It would be useful to determine drug loss in cell-free MPS for each of these compounds so that functional concentration of drug can be obtained.

The reviewer raises an important point, and we apologize for omitting this critical information addressing absorption of drugs and hydrophobic compounds, such as fatty acids, by PDMS[13]. As suggested, we determined the absorption of the drugs metformin, rosiglitazone, dexamethasone and semaglutide and fluorescent fatty acids in cell-free MPS. We didn't observe concentration changes of any of these drugs after 2 days of circulation, the treatment time used for all experiments. We show these data in the new Supplementary Figure 1b. Similarly, we observed absorption of fluorescent fatty acids by PDMS only after circulation for more than 4 days (Supplementary Figure 1a). We refer to these new data in lines 115-118 and 173-175 of the results section.

c. Related to comments 2 and 3, it would be very useful and supportive to have drug response data from MPS constructed with primary cells to use as a benchmark for the iPSC results.

We selected the insulin-sensitizing drugs metformin and rosiglitazone and the anti-inflammatory drug dexamethasone because their effects have been previously characterized in primary cells and patients[5–12], providing benchmarks for our experiments. As indicated in our response to the reviewer's comment 3 above, our results made in the MPS using our iPSC-derived cells are consistent with these benchmarks (Figure 3 and lines 258-271). Substantial literature also exists on the effects of semaglutide; however, because semaglutide is a newer drug, uncertainty still exists about the mechanistic basis of some of its effects. Of particular relevance to our study, most publications reported that hepatocytes are not a major semaglutide target, but contradictory findings have also been published[14–17]. To address this uncertainty, we first compared *GLP1R* gene expression between our iPSC-derived cells and primary cells, which produced equivalent results, with hepatocytes expressing the least amount of *GLP1R*. These new data support our finding that semaglutide specifically targets adipocytes in our MPS and are shown in the new Supplementary Figure 2.

To substantiate these results at the functional level, we performed tissue-specific semaglutide treatment within the context of the integrated M1-iADIPO-iHEP-MPS. These new experiments are shown in the revised Figure 4h-k. We found that selective semaglutide treatment of the M1-iADIPO-MPS antagonized MASLD in iHEP-MPS, as evidenced by reduced lipid accumulation and reversal of insulin resistance, for which we measured suppression of glucose production by insulin. In contrast, selective semaglutide treatment of the iHEP-MPS failed to reduce lipid accumulation and reverse insulin resistance, which was accompanied by increased levels of markers of inflammation, gluconeogenesis and lipogenesis in the iHEP-MPS. Together, these new data show that semaglutide prevents lipid accumulation and insulin resistance in hepatocytes by reducing WAT inflammation, acting specifically on adipocytes. Because these findings are consistent with clinical observations and data from mice, we believe that they serve as a sufficient replacement of experimental repeats using primary cells[18–20].

5. The use of iPSCs in MPS is a stepwise process, and an important limitation of this work is that the iHEP MPS is constructed with only iPSC-derived hepatocytes. Thus, a key point for the authors to discuss is that MASLD disease progression, including the development of steatosis and progression towards HIR involves multiple liver cell types (HSCs, LSECs, KCs), and that their current model may provide even more intricate analysis if these cell types are included in future iterations of the platform. This may be particularly useful in using the iADIPO-iHEP system as a drug development platform for more thoroughly evaluating the effects of GLP1R agonists like semaglutide in a liver model system that incorporates more of the relevant cell types.

We agree with the reviewer that increasing the cellular complexity of the iHEP-MPS is a worthwhile goal as it will allow for studies that address cellular interactions within the liver that

impact MASLD progression. As suggested by the reviewer, we added this future direction to the discussion section of our revised manuscript (lines 305-312).

• Minor weaknesses of this manuscript to address include:

1. In the Introduction (line 45) and subsequently throughout the rest of the manuscript, it should be noted that recently the terminology for metabolic-dysfunction associated fatty liver disease (MAFLD) has been changed to metabolic-dysfunction associated steatotic liver disease (MASLD) [PMID: 38223415]. It would be useful to change the terminology to reflect the change in nomenclature in this manuscript.

We changed MAFLD to MASLD throughout our revised manuscript.

2. In the Introduction (lines 61 and 62), the citations (refs 17 and 18) that reference the use of liver-on-a-chip models do not sufficiently reflect the role that these models have played in studying both MASLD and T2DM. There are multiple commercial and academic groups who have published MASLD/T2D-specific applications of their liver MPS platforms that should be described here so that the contributions of this manuscript can be put in better perspective.

We agree with the reviewer's point and expanded the introduction section (lines 70-91) in our revised manuscript, including additional references, to better capture the current state of liver-MPS for MASLD and T2DM modeling.

Reviewer #2 (Remarks to the Author):

In this study, the authors established the unique microphysiological system (MPS) that interconnects iPSC-differentiated isogenic adipocytes and hepatocytes. By co-culturing adipocytes with iPSC-derived macrophages, the authors also mimicked adipose tissue inflammation *in vitro* and reproduced the lipid influx from adipocytes to hepatocytes and hepatic insulin resistance found in fatty liver in patients with obesity and type 2 diabetes. Using this system, the authors tested directly the effect of a couple of anti-diabetic drugs such as metformin and rosiglitazone on lipid accumulation in iPSC-derived hepatocytes, and showed that a GLP-1RA semaglutide ameliorates lipid accumulation and insulin resistance in hepatocytes through the adipose-to-liver interaction. This study should provide clues to understand how the adipose tissue interacts with the liver, where lipid accumulation and insulin resistance occur during the development of obesity-induced adipose tissue inflammation, and even how some of antidiabetic drugs can act directly on the system to prevent and/or treat the diseased states. There are several concerns to be addressed.

We appreciate the reviewer's positive assessment of the value of the findings of our study and the potential of our system.

Major comments

1. Although this MPS is unique in that the authors could test the direct effect of chemicals/drugs on adipocytes, hepatocytes, and even macrophages within this system. The authors would pursue the mechanism underlying their observations. As the unique simplified *in vitro* model system, the authors would provide how the new findings obtained herein might be extrapolated to the more complex setting; using, for instance, mice with pharmacological intervention.

We appreciate the reviewer's recognition of the potential of our system. We also agree with the reviewer's point that putting our findings in context with more complex biological systems is desirable as it would increase mechanistic insight and establish the translational potential of our system. Therefore, we focused initially on testing prototypical insulin-sensitizing or anti-inflammatory drugs, which produced results that are consistent with findings by others made in mice or patients (Figure 3 and lines 258-271)[5–7,11,21,22].

In addition, we performed new experiments investigating the mechanisms of action of semaglutide, which is a newer drug and therefore less well characterized. Specifically, we determined where and how semaglutide acts on the adipose tissue-liver axis in MASLD development. For this, we performed tissue-specific semaglutide treatment within the context of our integrated M1-iADIPO-iHEP-MPS. These new experiments are shown in the revised Figure 4h-k. We found that selective semaglutide treatment of the M1-iADIPO-MPS antagonized MASLD in iHEP-MPS, as evidenced by reduced lipid accumulation and reversal of insulin resistance, for which we measured suppression of glucose production by insulin. In contrast, selective semaglutide treatment of the iHEP-MPS failed to reduce lipid accumulation and reverse insulin resistance, which was accompanied by increased levels of markers of inflammation, gluconeogenesis and lipogenesis in the iHEP-MPS. Together, these new data show that semaglutide prevents lipid accumulation and insulin resistance in hepatocytes by reducing WAT inflammation, acting specifically on adipocytes.

2. In the previous work, the authors reported that macrophage-derived inflammatory cytokines induce hepatic insulin resistance by inhibiting insulin signaling in iPSC-derived hepatocytes (**Nat Commun.** 2023). Given that macrophages are the upstream of hepatocytes in the new system,

macrophages-derived soluble factors may act directly on hepatocytes as well as adipocytes. The authors described transcriptional changes in hepatocytes, focusing on inflammation and glucose/lipid metabolism, which would, however, occur after the addition of soluble factors such as TNFα in hepatocytes. Unbiassed transcriptomic analysis in iHEP-MPS should be important.

The reviewer makes an important point raising the possibility that our findings of insulin resistance in the iHEP-MPS could simply be due to the presence of M1-iMACs in the M1-iADIPO-MPS, that is, independent of iADIPOs. To answer this question, we performed additional experiments shown in the new Extended Data Figure 2d-h and described in the results section (lines 157-165) of our revised manuscript. Briefly, we compared M1-iADIPO-iHEP-MPS to M1-iHEP-MPS, that is, interconnected MPS with and without iADIPOs, thereby defining the contribution of ADIPOs to the iHEP-MPS phenotype (Extended Data Figures 2d). We found that the presence of iADIPOs worsened the metabolic alterations in the iHEP-MPS, including glucose production at baseline and earlier onset of insulin resistance, for which we measured suppression of glucose production by insulin (Extended Data Figures 2e,f). These iADIPO-dependent alterations were accompanied by higher steatosis in the iHEP-MPS (Extended Data Figure 2g) and induced expression of genes reflecting inflammation, gluconeogenesis, lipogenesis, and lipid transport (Extended Data Figure 2h). This finding is consistent with our previous observation suggesting a proinflammatory loop between M1-iMACs and iADIPOs, which causes higher proinflammatory cytokine levels than M1-iMACs alone[23]. We thank the reviewer for prompting us to perform these additional experiments as they highlight the specific role adipocytes play in aggravating WAT inflammation and thus in the etiology of aberrations of glucose and lipid metabolism in hepatocytes.

3. A recent study reported the marked heterogeneity of adipocytes and macrophages in human adipose tissue, which might affect the adipose tissue phenotype (Nature 603:926-933, 2022). Are adipocytes and/or macrophages used relatively homogenous or phenotypically mixed in this system? Single cell/nucleus analysis of adipocytes in this system would be helpful.

The reviewer raises an interesting point about heterogeneity of cell types, which we added to the discussion of our revised manuscript, including the reference referred to by the reviewer (lines 300-312).

Regarding our iADIPOs, the robustness of our published differentiation protocol facilitates the production of a homogenous population of cells[3]. Their phenotype corresponds to that of the hAd3-like subcutaneous adipose tissue subpopulation described in the reference referred to by the reviewer, characterized by high *PNPLA3*, low *GALNT3*, absent *GRIA4* and low visceral adipose tissue marker gene expression[24].

As described in the methods section of our initial submission (lines 352-360 in revised manuscript), our published protocol for the generation of M1-iMACs includes a purification step to eliminate non-hematopoietic cells. The resulting population of cells is homogeneous as evidenced by analysis of multiple markers by flow cytometry in comparison to primary cells[2].

4. The authors stated that the GLP-1 analogue had subtle effects on macrophages and hepatocytes, based on the qPCR analysis of the cell types. Given the expression pattern of GLP-1R, however, I dare say that the GLP-1RA have considerable effect on hepatocytes and macrophages. The authors are required to verify how lipid accumulation in iHEP-MPS changes, when GLP-1 signaling is disrupted in adipocytes (e.g. knockdown of GLP-1R), so that they could suggest that the GLP-1RA acts more effectively on adipocytes than other cell types.

We agree with the reviewer and wrote in the results section of our initial submission (lines 190-192 in revised manuscript) that controversy exists about the cell types responding to semaglutide, including whether hepatocytes are targeted or not. To address this uncertainty, we first compared *GLP1R* gene expression between our iPSC-derived cells and primary cells, which produced equivalent results, with hepatocytes expressing the least amount of *GLP1R*. These new data support our finding that semaglutide specifically targets adipocytes in our MPS and are shown in the new Supplementary Figure 2.

To substantiate these results at the functional level, as described in our response to the reviewer's comment 1, we performed tissue-specific semaglutide treatment within the context of the integrated M1-iADIPO-iHEP-MPS. These new experiments are shown in the revised Figure 4h-k. We found that selective semaglutide treatment of the M1-iADIPO-MPS antagonized MASLD in iHEP-MPS, as evidenced by reduced lipid accumulation and reversal of insulin resistance, for which we measured suppression of glucose production by insulin. In contrast, selective semaglutide treatment of the iHEP-MPS failed to reduce lipid accumulation and reverse insulin resistance, which was accompanied by increased levels of markers of inflammation, gluconeogenesis and lipogenesis in the iHEP-MPS. Together, these new data show that semaglutide prevents lipid accumulation and insulin resistance in hepatocytes by reducing WAT inflammation, acting specifically on adipocytes.

Minor comment

5. The legends for Figure 2g and 2h do not correspond to Figures 2g and 2h.

We corrected the legends for Figures 2g,h in our revised manuscript (lines 753,754).

6. Line 140-141. Lipid accumulation in iHEP should be quantified in Figure 3a.

We quantified lipid accumulation in iHEPs in the revised Figure 3c.

Reviewer #3 (Remarks to the Author):

This is an interesting article that claims to develop the first human adipose tissue/liver MPS system with the inclusion of inactive and active monocytes. All three cell types have been derived from the same induced pluripotent stem cell line. They indicate that the presence of inflammation from the macrophages in the presence of the adipocytes cause lipid accumulation in the hepatocytes as well as insulin resistance. They monitored the systems response to two known therapeutics as well as a GLP1 receptor agonist that improved hepatocyte function by acting on the adipocytes.

While interesting, this paper has several major flaws that need to be addressed before publishing. First, they claim this is the first multi-organ hepatocyte/adipocyte MPS, which is false, as this was published by Slaughter et al. in Scientific Reports (2021). The authors knew this was false as they then reference this paper a few lines later to claim that it utilized just primary cells and cell lines are not as useful for this type of research, the latter statement also being false in that cell lines were not used in this paper and the primary cells were well-characterized first before being combined into the multi-organ system.

We acknowledge that Slaughter et al. were the first to report an adipocyte-hepatocyte MPS based on primary human cells and have revised our manuscript accordingly (lines 77-79). The work by Slaughter et al. provided an important steppingstone for our iPSC-based MPS using isogenic iADIPOs, iHEPs and iMACs.

In addition, primary cells if characterized properly are excellent models for in vitro experiments and can be useful for capturing population heterogeneity.

We agree with the reviewer and revised the introduction in our revised manuscript (lines 70-91) to better describe the advantages and disadvantages of both primary and iPSC-derived cells in the context of MPS development.

In addition, most of Figure 2 simply repeats the results from the Slaughter paper and they utilized metformin as one of the drugs that was also in the Scientific Reports paper, which indicated that it was only effective in the MPS model at supraphysiological concentrations and the reason why this drug may not be effective clinically. No mention of this is made in this paper, and as a matter of fact, concentrations for the drugs are not listed in the figures or text and there is no section in the Methods that describes drug administration.

Naturally, some experimental designs in our study are inspired by previous pioneering work such as the paper by Slaughter et al. 2021. In fact, we selected the insulin-sensitizing drugs metformin and rosiglitazone and the anti-inflammatory drug dexamethasone because their effects have been previously characterized in primary cells and patients[5–12], providing benchmarks for our experiments using iPSC-derived cells. A substantial difference of our study to previous studies is that ours focuses on WAT inflammation, for which we introduced M1 iMACs into the iADIPO-MPS. Consequently, many of our analyses address inflammation and its consequences, including disease-driving insulin resistance, which has not been done in the past.

The concentrations of all drugs were listed under “MPS culture” in the methods section of our initial submission (lines 363-365). We recognize that this was not a good placement and have added a new section, “Drug administration”, to the methods section (lines 465-473) in our revised manuscript. In addition, we have added the drug concentrations to the main text (lines 170,171, 192, 208).

We used high drug concentrations, for example, metformin at 10 mM, to be able to limit the circulation time in the integrated M1-iADIPO-iHEP-MPS to 2 days. We derived these concentrations from previous studies using primary cells[9,25,26] to be able to benchmark our iPSC-derived cell-based system as is now made clear in the revised manuscript (lines 269-271).

A highlight of this paper is using all three cell types from the same stem cell line, but to some degree, this defeats the argument that primaries are a bad model because of their heterogeneity. Whereas it is possible that their pluripotent stem cells are derived from cells from a high performing outlier and they would not know this unless they did multiple cell lines. The paper does make important points for insulin resistance as well as possible therapeutics that block adipocyte to hepatocyte communication which could be a significant finding.

We thank the reviewer for making this important point. We previously compared iADIPOs, iHEPs, and iMACs generated from the iPSC line we used in our study (WTC, GM25256) to primary cells and derivatives of other iPSC lines[1–4]. Our data indicate that our optimized differentiation protocols are effective in generating from many different iPSC lines iADIPOs, iHEPs, and iMACs that are similar to primary cells in differentiation and function. We used the WTC line in this study because it stems from a healthy donor and has been fully sequenced (https://www.cellosaurus.org/CVCL_Y803).

To be acceptable, the paper would have to correct the untrue statements by properly referencing the original paper and highlighting the differences to establish a strong premise for the work to be published in a high-level journal. To also strengthen the premise, they should reference additional publications on multi-organ MPS for other indications to highlight the significance and uniqueness of their approach.

We revised the presentation of the pioneering work by Slaughter et al. 2021 using a primary cell-based MPS in the introduction and discussion sections (lines 77-79, 269-271, 300-312) of our revised manuscript, including a detailed description of the advantages and disadvantages of both primary and iPSC-derived cells in the context of MPS development. In addition, we added a paragraph to the introduction section (lines 70-91), including additional references, to better capture the current state of liver-MPS for MASLD and T2DM modeling, thereby defining the advances made by our study.

Reviewer #4 (Remarks to the Author):

I co-reviewed this manuscript with one of the reviewers who provided the listed reports. This is part of the Nature Communications initiative to facilitate training in peer review and to provide appropriate recognition for Early Career Researchers who co-review manuscripts.

We thank the co-reviewer for their effort.

References

1. Lee-Montiel, F.T. et al. Integrated Isogenic Human Induced Pluripotent Stem Cell-Based Liver and Heart Microphysiological Systems Predict Unsafe Drug-Drug Interaction. *Front Pharmacol* **12**, 667010 (2021).
2. Matsuo, K. et al. ACVR1R206H extends inflammatory responses in human induced pluripotent stem cell-derived macrophages. *Bone* **153**, 116129 (2021).
3. Qi, L. et al. Probing Insulin Sensitivity with Metabolically Competent Human Stem Cell-Derived White Adipose Tissue Microphysiological Systems. *Small* **18**, e2103157 (2021).
4. Groeger, M. et al. Modeling and therapeutic targeting of inflammation-induced hepatic insulin resistance using human iPSC-derived hepatocytes and macrophages. *Nat Commun* **14** (2023).
5. Bai, B. & Chen, H. Metformin: A Novel Weapon Against Inflammation. *Front Pharmacol* **12**, 622262 (2021).
6. Coutinho, A.E. & Chapman, K.E. The anti-inflammatory and immunosuppressive effects of glucocorticoids, recent developments and mechanistic insights. *Mol Cell Endocrinol* **335**, 2–13 (2011).
7. Juurinen, L., Kotronen, A., Granér, M. & Yki-Järvinen, H. Rosiglitazone reduces liver fat and insulin requirements and improves hepatic insulin sensitivity and glycemic control in patients with type 2 diabetes requiring high insulin doses. *J Clin Endocrinol Metab* **93**, 118–124 (2008).
8. Le Pelletier, L. et al. Metformin alleviates stress-induced cellular senescence of aging human adipose stromal cells and the ensuing adipocyte dysfunction. *Elife* **10** (2021).
9. Oh, H.-Y. et al. Dexamethasone protects primary cultured hepatocytes from death receptor-mediated apoptosis by upregulation of cFLIP. *Cell Death Differ* **13**, 512–523 (2006).
10. Slaughter, V.L. et al. Validation of an adipose-liver human-on-a-chip model of NAFLD for preclinical therapeutic efficacy evaluation. *Sci Rep* **11**, 13159 (2021).
11. Tiikkainen, M. et al. Effects of rosiglitazone and metformin on liver fat content, hepatic insulin resistance, insulin clearance, and gene expression in adipose tissue in patients with type 2 diabetes. *Diabetes* **53**, 2169–2176 (2004).
12. Fryklund, C., Morén, B., Neuhaus, M., Periwal, V. & Stenkula, K.G. Rosiglitazone treatment enhances intracellular actin dynamics and glucose transport in hypertrophic adipocytes. *Life Sci* **299**, 120537 (2022).
13. Toepke, M.W. & Beebe, D.J. PDMS absorption of small molecules and consequences in microfluidic applications. *Lab Chip* **6**, 1484–1486 (2006).
14. Fang, Y. et al. Liraglutide Alleviates Hepatic Steatosis by Activating the TFEB-Regulated Autophagy-Lysosomal Pathway. *Front Cell Dev Biol* **8**, 602574 (2020).
15. Gupta, N.A. et al. Glucagon-like peptide-1 receptor is present on human hepatocytes and has a direct role in decreasing hepatic steatosis in vitro by modulating elements of the insulin signaling pathway. *Hepatology* **51**, 1584–1592 (2010).

16. Panjwani, N. et al. GLP-1 receptor activation indirectly reduces hepatic lipid accumulation but does not attenuate development of atherosclerosis in diabetic male ApoE(-/-) mice. *Endocrinology* **154**, 127–139 (2013).

17. Yabut, J.M. & Drucker, D.J. Glucagon-like Peptide-1 Receptor-based Therapeutics for Metabolic Liver Disease. *Endocr Rev* **44**, 14–32 (2023).

18. Garvey, W.T. et al. Two-year effects of semaglutide in adults with overweight or obesity: the STEP 5 trial. *Nat Med* **28**, 2083–2091 (2022).

19. Niu, S. et al. Semaglutide ameliorates metabolism and hepatic outcomes in an NAFLD mouse model. *Front Endocrinol (Lausanne)* **13**, 1046130 (2022).

20. Lee, Y.-S. et al. Glucagon-like peptide-1 inhibits adipose tissue macrophage infiltration and inflammation in an obese mouse model of diabetes. *Diabetologia* **55**, 2456–2468 (2012).

21. Wang, S., Goodspeed, L., Turk, K.E., Houston, B. & Hartigh, L.J. den Rosiglitazone Improves Insulin Resistance Mediated by 10,12 Conjugated Linoleic Acid in a Male Mouse Model of Metabolic Syndrome. *Endocrinology* **158**, 2848–2859 (2017).

22. Yang, L. et al. Metformin inhibits inflammatory response and endoplasmic reticulum stress to improve hypothalamic aging in obese mice. *iScience* **26**, 108082 (2023).

23. Qi, L. et al. Human iPSC-Derived Proinflammatory Macrophages cause Insulin Resistance in an Isogenic White Adipose Tissue Microphysiological System. *Small* **19**, e2203725 (2023).

24. Emont, M.P. et al. A single-cell atlas of human and mouse white adipose tissue. Nature 603, 926–933 (2022).

25. Merlin, J. et al. The PPARγ agonist rosiglitazone promotes the induction of brite adipocytes, increasing β-adrenoceptor-mediated mitochondrial function and glucose uptake. *Cell Signal* **42**, 54–66 (2018).

26. Luizon, M.R. et al. Genomic Characterization of Metformin Hepatic Response. *PLoS Genet* **12**, e1006449 (2016).

REVIEWERS' COMMENTS

Reviewer #2 (Remarks to the Author):

None

Reviewer #3 (Remarks to the Author):

1) Cellular dynamics within the proposed MPS is an interesting point of this work. Although, Slaughter et al. was refernced to identify the fundamental importance of tissue dynamims, the authors did not reference the significance of the integration of the different blood mimetic mediums and their influence on cellular response. Specifically, the Slaughter system utilized serum-free media formulations tailored to represent different human metabolic states—healthy, corresponding to normal human postprandial glucose and insulin concentrations, diabetic, with representative glucose and insulin, obese, with representative recirculating free fatty acids (FFA).

2) Have the authors quantified the concentrations of the drug in culture over various timepoints during the study to determine the exact drug exposure over time?

This article can be accepted after major revisions.

Reviewer #4 (Remarks to the Author):

All reviewer points were thoughtfully addressed, including the addition of new experimental data which strengthens the overall conclusions of this paper.

REVIEWERS' COMMENTS

Reviewer #2 (Remarks to the Author):
None

Author response: We thank the reviewer for their comments guiding us to maximize the impact and clarity of our manuscript.

Reviewer #3 (Remarks to the Author):
1) Cellular dynamics within the proposed MPS is an interesting point of this work. Although, Slaughter et al. was refernced to identify the fundamental importance of tissue dynamims, the authors did not reference the significance of the integration of the different blood mimetic mediums and their influence on cellular response. Specifically, the Slaughter system utilized serum-free media formulations tailored to represent different human metabolic states—healthy, corresponding to normal human postprandial glucose and insulin concentrations, diabetic, with representative glucose and insulin, obese, with representative recirculating free fatty acids (FFA).

Author response: The reviewer makes an important point about the use of defined media to model metabolic states in the paper by Slaughter et al., which we added to the introduction of our revised manuscript (line 69).

2) Have the authors quantified the concentrations of the drug in culture over various timepoints during the study to determine the exact drug exposure over time?

Author response: Our new data in cell-free MPS included in the revised manuscript show that the drugs we used are stable in solution and that there is no absorption by PDMS during the 48-hour circulation period (Supplementary Fig. S1b). Of all drugs we used, the long-chain fatty acid (C1-fluorophore-C12) is expected to be the most absorbed by PDMS because of its high lipophilicity. When we investigated the concentration of C1-fluorophore-C12 over time, we found no significant change up to 4 days of circulation, which excludes absorption by PDMS within the 2-day experimental period, not only for this drug but also for the other drugs because they are less lipophilic than C1-fluorophore-C12.

Reviewer #4 (Remarks to the Author):
All reviewer points were thoughtfully addressed, including the addition of new experimental data which strengthens the overall conclusions of this paper.

Author response: We thank the reviewer for their comments guiding us to maximize the impact and clarity of our manuscript.
